# Synergistic osteogenesis, angiogenesis, and immune reprogramming by a metal-phenolic functionalized electrospun fibrous membrane for alveolar bone regeneration

**DOI:** 10.1016/j.mtbio.2026.103045

**Published:** 2026-03-18

**Authors:** Jia Zhou, Yue Hu, Jiali Bao, Shiyuan Yang, Yan Zhu, Zixiao Zhang, Minxi Chen, Yuning Zhou, Kaili Lin, Yuanjin Xu

**Affiliations:** a Department of Oral Surgery, Shanghai Ninth People's Hospital, Shanghai Jiao Tong University School of Medicine, Huangpu District, Shanghai, China; b College of Stomatology, Shanghai Jiao Tong University, National Center for Stomatology, National Clinical Research Center for Oral Diseases, Shanghai Key Laboratory of Stomatology, Shanghai Research Institute of Stomatology, Huangpu District, Shanghai, China; c Department of Oral and Cranio-maxillofacial Surgery, Shanghai Ninth People's Hospital, Shanghai Jiao Tong University School of Medicine, Huangpu District, Shanghai, China

**Keywords:** Electrospinning, Alveolar bone regeneration, Curcumin, Metal-phenolic nanoparticles, Osteogenesis

## Abstract

Alveolar bone defects, whether caused by pathology or trauma, severely compromise oral function and facial aesthetics. However, current commercial guided bone regeneration (GBR) membranes exhibit limitations in mechanical properties, degradation rate matching, and osteogenic activity. In response to these challenges, this study developed a multifunctional bioactive electrospun fibrous membrane (EFM) composed of silk fibroin and polycaprolactone and loaded with curcumin-strontium nanoparticles (Cur-Sr/SF/PCL). This membrane demonstrates superior mechanical strength, suitable degradation behavior, and effective barrier function against soft tissue infiltration. Crucially, the synergistic release of curcumin and Sr^2+^ collectively promotes osteogenic differentiation, enhances angiogenic capacity, and modulates the immune microenvironment, ultimately facilitating alveolar bone regeneration. Mechanistic analysis has further revealed that the pro-osteogenic effect of the Cur-Sr/SF/PCL EFM could be mediated by the initiation of the Wnt/Ca^2+^/calcineurin (CaN) signaling cascade. In summary, the Cur-Sr/SF/PCL EFM designed in this study synergistically promotes bone regeneration through multiple biological mechanisms, thus providing a promising and novel GBR approach for alveolar bone defect repair.

## Highlights

• Cur-Sr/SF/PCL has favorable mechanical strength, flexibility, and adaptability.
• Cur-Sr/SF/PCL exhibits a suitable degradation rate and a firm barrier function.
• Cur-Sr/SF/PCL exerts osteogenic, angiogenic, immunomodulatory capacities.
• Cur-Sr/SF/PCL promotes osteogenesis via the Wnt/Ca^2+^/CaN signaling pathway.
• Cur-Sr/SF/PCL elicited notable bone regeneration in rat alveolar bone defects.

## Graphical abstract

**Figure:**
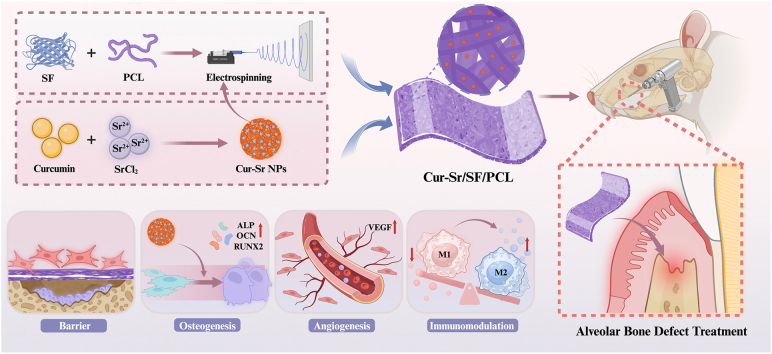

## 1 Introduction

Alveolar bone defects are prevalent oral conditions, frequently associated with periodontal diseases, trauma, or long-term tooth loss [1,2]. This loss of bone volume not only compromises facial aesthetics and masticatory function but also jeopardizes the success of subsequent implant rehabilitation [3,4]. However, a key biological challenge in restoring alveolar bone defects lies in the innate periodontal healing process, where gingival epithelial and connective tissues exhibit a proliferation rate outpacing that of alveolar bone tissues [5]. This competitive growth often results in the formation of a long junctional epithelium and deep periodontal pockets, ultimately impeding effective bone regeneration [6].

Guided bone regeneration (GBR) currently stands as a predominant clinical approach for the reconstruction of alveolar bone deficiencies [7]. Placing a GBR barrier between soft tissues and bone defects prevents epithelial cells and fibroblasts from invading into the osteogenic zone, thereby creating a microenvironment conducive to osteoblast proliferation and subsequent bone regeneration [8,9]. To fulfill the demands of clinical application, an optimal GBR membrane is required to exhibit favorable biocompatibility, sufficient space maintaining capacity, and an appropriate degradation rate [10,11]. Nevertheless, currently available GBR membranes employed in clinical practice still exhibit several limitations. For example, the Bio-Gide^Ⓡ^ collagen membrane, one of the most extensively utilized GBR membranes in clinical practice, is limited by a relatively rapid degradation rate and diminished mechanical properties in a moist environment. These shortcomings may predispose it to collapse within the bone defect, thereby compromising its space maintaining capacity and ultimately leading to suboptimal osteogenesis [12,13]. Furthermore, currently available clinical GBR membranes primarily serve as physical barriers during the regeneration process and exhibit a distinct lack of osteoinductivity [14]. Therefore, to further enhance the therapeutic efficacy of GBR, it is of significant clinical importance to develop novel GBR membranes that possess proper degradation rate synchronized with the pace of bone regeneration, superior mechanical strength, and the ability to induce bone regeneration. The crucial contribution of orofacial mesenchymal stem cells (OMSCs) to alveolar bone repair has been well-established [15]. Meanwhile, sufficient vascular supply within the osseous defect site is a fundamental requirement for successful bone reconstruction [16]. Consequently, an optimal GBR membrane must be fabricated to simultaneously enhance osteogenic differentiation of OMSCs while facilitating angiogenesis in the defect region.

Moreover, the immune microenvironment within the bone injury region exhibits significant dysregulation due to persistent inflammatory responses triggered by bacterial invasion, necrotic tissue and mechanical irritation [17]. These local alterations can polarize macrophages into pro-inflammatory M1 macrophages and anti-inflammatory M2 macrophages [18,19]. Studies indicate that M1 macrophages are swiftly mobilized to the injury site, followed by a programmed switch to the M2 phenotype typically occurring within 3-4 days post-injury [20]. However, when macrophages fail to execute this transition from M1 to M2 phenotype, it compromises the prompt clearance of inflammation and tissue repair, ultimately leading to impaired bone regeneration [21]. Hence, the ability of GBR membranes to orchestrate macrophage polarization is essential for successful alveolar bone defect repair.

Electrospinning technology is the most widely utilized approach for developing novel GBR membranes. This preference is attributed to the high specific surface area and elevated porosity characteristic of electrospun fibrous membranes (EFMs). These attributes enable EFMs to replicate structural characteristics of native extracellular matrix (ECM), thereby creating a biomimetic architecture that facilitates cellular attachment, proliferation, and differentiation[[22], [23], [24]]. Among the various materials available for electrospinning, polycaprolactone (PCL) is a prominent biodegradable polymer FDA-approved for medical applications, renowned for its excellent biocompatibility and mechanical properties[13,[25], [26], [27]]. Furthermore, owing to its minimal production of acidic byproducts during hydrolysis, PCL is extensively used in specialized drug delivery platforms, *in vivo* device implantation and regenerative medicine [28,29]. However, PCL also presents certain limitations, such as a notably protracted degradation rate, inadequate surface hydrophilicity, and suboptimal cellular attachment [7]. Consequently, this study aims to fabricate a blended EFM by combining PCL with other polymeric materials to achieve complementary advantages and overcome these drawbacks.

As a highly purified protein derived from silk, silk fibroin (SF) comprises various amino acids and the end products of its decomposition are absorbable by the human body, consisting of amino acids or oligopeptides. This feature endows it with superior biocompatibility, oxygen permeability, and biodegradability, while eliciting only a mild inflammatory response upon implantation [30,31]. Notably, SF has become an attractive biomaterial for bone regeneration strategies, largely because its structure shares similarities with type I collagen, the predominant organic constituent of native bone [32,33]. These properties position SF as an excellent candidate for developing GBR membranes, offering a potential solution to the limitations of current materials. Therefore, this study aims to integrate the advantages of PCL and SF to fabricate a SF/PCL EFM. The fabricated membrane is expected to exhibit superior mechanical properties, favorable biocompatibility, an appropriate degradation rate, and the ability to facilitate osteoinduction.

Based on previous work from our research group and extensive literature reports, we have conducted in-depth and comprehensive research on natural polyphenolic compounds [2,13,15,34]. Curcumin, the main active component from turmeric, is a natural polyphenol with diverse pharmacological activities, including osteoinductive, angiogenic, antioxidant, anti-inflammatory, and immunomodulatory effects[[35], [36], [37], [38]]. However, the burst or uncontrolled sustained release of the drug may lead to an excessively high local drug concentration, which could conversely interfere with the bone regeneration process [39]. Besides, curcumin possesses extremely low water solubility and poor bioavailability, which significantly limits its application. Therefore, employing nanocarriers to load curcumin for constructing a sustained-release drug delivery system can not only preserve its bioactivity and enhance its bioavailability, but also achieve controlled drug release, ultimately attaining the desired therapeutic efficacy [40].

A notable advantage of curcumin lies in the phenolic hydroxyl groups within its molecular structure, which confer a robust capacity to coordinate with various metal ions, including Cu^2+^ and Mg^2+^, thereby forming metal-phenolic networks (MPNs) with diverse biomedical applications [37,41]. Numerous studies have reported that metal-coordinated curcumin nanoparticles significantly enhance its bioavailability and enable sustained drug release[[42], [43], [44], [45]]. Among these metal ions, extensive international and domestic research, including our group's prior work, has demonstrated that strontium ions (Sr^2+^) exhibit dual functionality in bone metabolism by promoting osteogenesis and inhibiting osteoclastogenesis, as well as possessing potent pro-angiogenic capabilities [46,47].

**Scheme 1:**
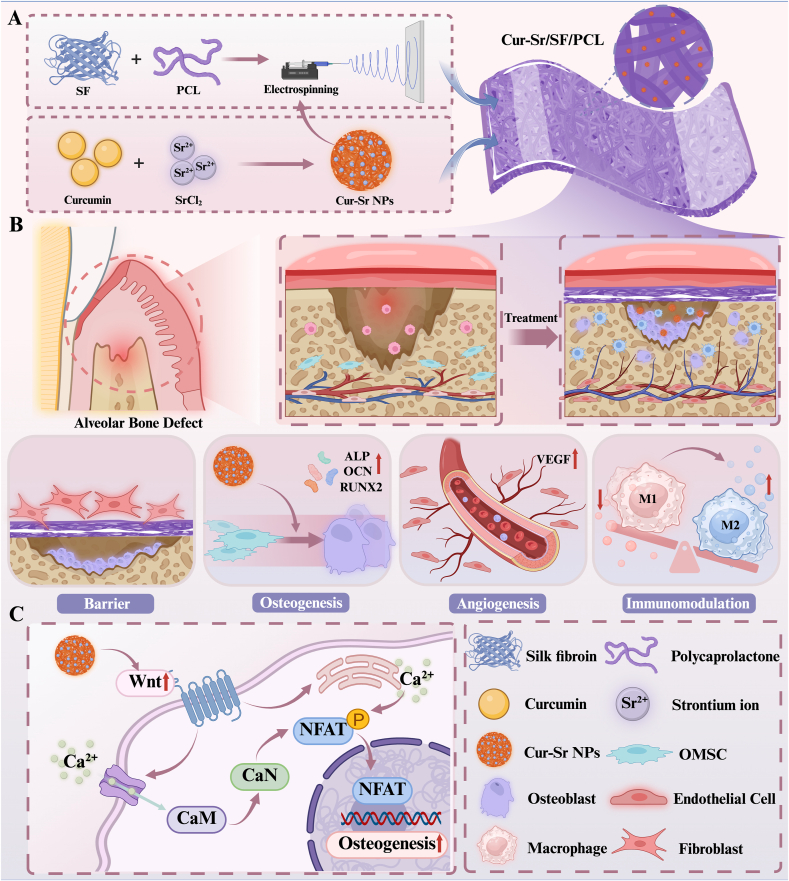
Illustration of the Cur-Sr/SF/PCL EFM for alveolar bone regeneration. A) The preparation of the Cur-Sr/SF/PCL EFM. B) The Cur-Sr/SF/PCL EFM promoted alveolar bone regeneration via barrier function, osteogenesis, angiogenesis and immunomodulation. C) The Cur-Sr/SF/PCL EFM enhanced osteogenesis by activating the Wnt/Ca^2+^/CaN signaling pathway.

In this study, a composite EFM of SF and PCL incorporated with Cur-Sr nanoparticles (Cur-Sr NPs) was fabricated via electrospinning. Both *in vitro* experiments and *in vivo* studies utilizing a rat alveolar bone defect model demonstrate that this novel GBR membrane exhibits excellent biocompatibility, desirable mechanical properties, and a synchronized degradation rate. Additionally, the potential for large-scale production and low cost is attributable to the straightforward electrospinning process and the cost-effectiveness of readily available curcumin raw material. Moreover, the Cur-Sr/SF/PCL EFM demonstrates a synergistic effect by promoting osteogenesis, enhancing angiogenesis, and modulating macrophage polarization, thereby underscoring its pontential as a promising therapeutic strategy for alveolar bone restoration (Scheme 1).

## 2 Materials and methods

### 2.1 Materials

Curcumin (≥98.0%) was acquired from Sigma-Aldrich Co. (USA), Strontium chloride (≥99.5%), PCL (Mn = 80000), polyvinylpyrrolidone (PVP, Mn = 8000), hexafluoroisopropanol (HFIP, ≥99.5%) were provided by Aladdin Co., Ltd. (Shanghai, China).

### 2.2 Synthesis of the Cur-Sr NPs

The Cur-Sr NPs were synthesized following an established protocol with slight adjustments [44]. In short, we solubilized 66 mg of PVP in 5 mL of methanol under stirring. Next, a 1 mL methanol mixture containing 20 mg of SrCl_2_ was introduced drop by drop into the PVP solution, followed by stirring for 30 min. Then, 1 mL of a methanolic curcumin solution (10 mg) was slowly dropped in, and the resulting blend was allowed to stir for another 3 h. Following a 48-h dialysis period, the Cur-Sr NPs were finally harvested for subsequent applications.

### 2.3 Preparation of SF

SF was extracted according to an established method [33]. To summarize, silkworm (*Bombyx mori*) cocoons were subjected to boiling in a 0.02 M Na_2_CO_3_ solution for 30 min to strip the sericin. The degummed cocoons were then thoroughly rinsed with distilled water three times (30 min each) to eliminate residual reagents, followed by drying for over 12 h. We then solubilized the dry fibroin in a 9.3 M LiBr solution. The final preparation underwent dialysis (MW CO= 3500 Da, Yeasen, China) over a 48-h period, preceding centrifugation for the separation of any insoluble aggregates. Finally, the purified SF solution was lyophilized for subsequent applications.

### 2.4 Preparation of the EFMs

Appropriate amounts of curcumin or Cur-Sr NPs were weighed and added to HFIP, followed by sonication for 30 min [13]. PCL and SF were then added to this solution at a mass ratio of 8:2 (PCL: SF) to yield a 10% (w/v) solution system. The electrospinning operation proceeded using an applied voltage of ±10 kV, with a solution feed rate of 0.6 mL/h, a collection distance of 15 cm, and a collector rotation speed of 10 rpm. The electrospinning process continued for 4 h to obtain Cur/SF/PCL and Cur-Sr/SF/PCL EFMs. Corresponding SF/PCL EFMs were prepared as a control group. The resulting membranes had an average thickness of approximately 60 μm, were cut into specific-sized (14 mm or 33 mm) circular discs, and subjected to ultraviolet sterilization before subsequent applications. The detailed electrospinning parameters are summarized in Table S1.

### 2.5 Characterization of the Cur-Sr NPs

The morphology of the Cur-Sr NPs was examined via scanning electron microscopy (SEM) (ZEISS GeminiSEM 300, German) and transmission electron microscopy (TEM) (JEOL JEM-2100F, Japan). The particle size distribution of the nanoparticles underwent statistical analysis utilizing ImageJ software. Additionally, the chemical composition of the Cur-Sr NPs was examined by fourier transform infrared spectroscopy (FTIR) (Thermo Fisher Scientific Nicolet iS20, USA) and X-ray photoelectron spectroscopy (XPS) (Thermo Scientific K-Alpha, USA). Drug content of the Cur-Sr NPs was quantitatively determined using thermogravimetric analysis (TGA) (TA Q500, USA).

### 2.6 Characterization of the EFMs

The external appearance of the EFMs were investigated utilizing SEM. The average nanofiber diameter was calculated by randomly assessing 100 individual fibers from representative SEM images using ImageJ software. The molecular composition of the membranes was elucidated via FTIR. The water contact angle was measured on membranes using a contact angle goniometer (Dingshen JY-82, China) to assess their surface hydrophilicity [13].

To evaluate the degradation rate, the EFMs were sectioned into rectangular specimens (3 cm × 5 cm). The initial weight (W_0_) of every specimen was documented. These specimens were then submerged in 10 mL of phosphate-buffered saline (PBS) and incubated in a 37 °C shaker for a period of 56 days. At specific time points, specimens were desiccated and weighed again (denoted as W_t_). The percentage degradation was determined via the formula: Degradation rate (%) = [(*W*_*0*_ - *W*_*t*_*)*/*W*_*0*_] × 100% [25]. For drug release study, the EFMs were sectioned into circular pieces (14 mm in diameter) and immersed in PBS within a 37 °C shaker [13]. The cumulative drug release was measured by determining the drug concentration within the supernatant at predetermined time intervals using UV-Vis spectrophotometry at 425 nm [17].

We use a universal testing machine (Hengyi, China) to evaluate tensile mechanical characteristics. Membranes were sectioned into rectangular specimens of identical dimensions (3 cm × 5 cm), and the thickness of each specimen was recorded. Subsequently, specimens were then secured between dual sensors and tested under a 500 N tensile force at 5 mm/min [27].

### 2.7 Isolation of OMSCs

Every animal experiment was conducted strictly adhering to the guidelines granted by the Institutional Animal Care and Use Committee of Shanghai Ninth People's Hospital, Shanghai Jiao Tong University School of Medicine (Shanghai, China) (Approval No. SH9H-2024-A99-1). Four-week-old Sprague-Dawley (SD) rats were euthanized, and mandibles were aseptically dissected out. OMSCs were then obtained and cultured with α-MEM medium containing 10% fetal bovine serum (FBS) and 1% penicillin/streptomycin. OMSCs at passages 3-6 were selected for subsequent experiments [15].

### 2.8 Isolation of human gingival fibroblasts (HGFs)

Ethical clearance for this study was granted by the Medical Ethical Committee of Shanghai Ninth People's Hospital affiliated to Shanghai Jiao Tong University School of Medicine (Approval No. SH9H-2022-T370-1). HGFs were derived from gingival tissues obtained during the removal of impacted third molars in patients between 18 and 25 years old. The gingival tissues were minced using sterile ophthalmic scissors, covered with coverslips, and cultured with complete α-MEM medium. HGFs at passages 3-6 were chosen for subsequent experiments [48].

### 2.9 Cytocompatibility evaluation of the EFMs

To evaluate the influence of the EFMs on cellular propagation, the Cell Counting Kit-8 (CCK-8) assay was utilized. Membranes were trimmed into circular pieces (14 mm in diameter) and firmly secured to the base of 24-well plates. OMSCs, HGFs, Human Umbilical Vein Endothelial Cells (HUVECs), and RAW264.7 cells were plated onto membranes at a density of 1 × 10^4^ cells per well. After 1, 3, and 5 days of incubation, the CCK-8 working solution (Beyotime, C0039, China) was added into every well, followed by a 60-min incubation at 37 °C. Subsequently, the resulting optical density of the supernatant was read at 450 nm using a Synergy H1 microplate reader (BioTek, USA). A live/dead assay was conducted for OMSCs and HGFs cultured on the membranes after 1 and 3 days. A working solution (Dojindo, C542, Japan) was dispensed into each well and allowed to react at 37 °C. Micrographs were acquired via a confocal laser scanning microscope (CLSM) (Nikon, Japan) [13].

To visualize adherence and appearance of cells on the EFMs, OMSCs and HGFs cultured for 1 and 3 days were subjected to staining for nuclei and cytoskeleton. Fixed and permeabilized, the samples were subsequently exposed to 100 nM phalloidin (Yeasen, 40734ES75, China) solution for 50 min. Afterward, nuclei material was counterstained using 4′,6-diamidino-2-phenylindole (DAPI) (Beyotime, C1006, China). Lastly, micrographs were acquired via a CLSM (Zeiss, German) [49].

### 2.10 Barrier function of the EFMs

The barrier capability was characterized by assessing the infiltration of HGFs. HGFs were plated onto the membranes at a density of 1 × 10^4^ cells per well and cultured for 4 and 7 days. Subsequently, cytoskeletal staining was performed to visualize the cells. Images were acquired using a CLSM (Zeiss, German), followed by 3D reconstruction to evaluate the penetration of HGFs [17].

### 2.11 *In vitro* osteogenic induction capacity of the EFMs

The *in vitro* osteogenic induction capacity was evaluated by alkaline phosphatase (ALP) staining, Alizarin Red S (ARS) staining, and the detection of osteogenesis-associated genes and proteins [13,26,27]. The membranes were seeded with OMSCs at a concentration of 1 × 10^4^ cells per well. After 4 and 7 days of culture, ALP staining was carried out and the ALP activity of OMSCs was quantitatively measured. After culturing for 28 days, mineralization nodules were stained with an ARS solution (Solarbio, G1452, China). The prepared samples were then examined and photographed via a stereomicroscope (Nikon, Japan).

OMSCs were cultured on membranes for 7 days. RNA was extracted from the cells and subsequently converted into cDNA. Quantitative real-time PCR (RT-qPCR) was then executed using a TB Green Premix Ex Taq kit (Takara, RR420A, Japan) to measure the expression level of osteogenesis-related genes. The primer sequences used can be found in Table S2.

OMSCs were seeded onto the membranes at a density of 1 × 10^4^ cells per well and cultured for 7 days. Fixed and permeabilized, the cells were treated by exposure to QuickBlock™ Blocking Buffer for Immunostaining (Beyotime, P0260, China) to mitigate non-specific binding. The cells were then submerged overnight at 4 °C in primary antibodies against runx family transcription factor 2 (RUNX2) and vascular endothelial growth factor (VEGF), respectively. The following day, the cells were incubated for 1 h with the secondary antibody. Finally, the nuclei and cytoskeleton were counterstained. We used a CLSM (Nikon, Japan) to visualize samples.

After 14 days of culture on the membranes, OMSCs were lysed to extract total protein. Subsequently, the expression level of the RUNX2, osteopontin (OPN) and osteocalcin (OCN) proteins were assessed via Western blotting. The antibodies used are listed in Table S3.

### 2.12 Recruitment effect of the EFMs

The capacity of cells to vertically migrate induced by the EFMs was evaluated using a Transwell cell culture system (Labselect, 14341, China, 8 μm). OMSCs (1 × 10^5^ cells per well) and HUVECs (2 × 10^4^ cells per well) were plated into the upper chamber with FBS-free medium, while the lower chamber contained complete medium and various EFMs. Following a 24-h incubation period, the migrated cells on the filter membrane's lower surface were subjected to crystal violet staining. Finally, a stereomicroscope (Nikon, Japan) was used to observe stained cells [49].

A scratch assay was conducted to evaluate the influence of the EFMs on horizontal migratory capacity of HUVECs. HUVECs were seeded at a concentration of 1 × 10^4^ cells per well and allowed to adhere for 12 h. A uniform scratch line was then generated across the HUVECs cell layer, and the initial images (0-h time point) were acquired using an inverted microscope (Nikon, Japan). Subsequently, 500 μL of material extracts (prepared by immersing the EFMs in FBS-free culture for 48 h) was introduced to each well. The cells continued incubation for an additional 24 h and were captured again to assess cell migration into the scratched area [27].

### 2.13 *In vitro* angiogenic function of the EFMs

The *in vitro* function of the EFMs to promote angiogenesis was assessed by a tube formation assay. Briefly, pre-cooled tips were used to coat 24-well plates with 250 μL of Matrigel (Yeasen, 40186, China), which was then solidified at 37 °C. HUVECs (1.5 × 10^5^ cells per well) were seeded on Matrigel and treated with 1 mL of material extracts (obtained by 48-h immersion of the EFMs in complete culture). Following 6 h of incubation, the tube-like networks were observed via an inverted microscope and quantified with ImageJ [27].

After OMSCs and HUVECs were cultured on the EFMs for 7 days and 4 days, respectively, the expression of angiogenesis-related genes and proteins was determined by RT-qPCR and immunofluorescence staining. The primer sequences and antibodies used can be found in Tables S2 and S3.

### 2.14 *In vitro* immune reprogramming effect of the EFMs

RAW 264.7 cells were seeded onto the EFMs at a density of 1 × 10^4^ cells/well and stimulated with lipopolysaccharide (LPS) (Solarbio, L8880, China) at a final concentration of 1 μg/mL to induce macrophage polarization [34]. After 24 h of LPS treatment, the expression of M1-related and M2-related genes and proteins was assessed by RT-qPCR and immunofluorescence staining. The primer sequences and antibody used can be found in Tables S2 and S3.

### 2.15 RNA sequencing and analysis

To comprehensively analyze the transcriptomic changes induced by the EFMs, OMSCs were cultured on the membranes for 7 days and total RNA was then extracted from the harvested cells. The RNA samples were submitted to LIANCHUAN Biotechnology Co., Ltd. (Hangzhou, China) for RNA sequencing (RNA-Seq) and subsequent data analysis. To confirm the findings from the transcriptomic analysis, Western blotting was performed to determine the protein levels of key signaling pathway components. All antibodies used are detailed in Table S3.

### 2.16 *In vivo* therapeutic effect in a rat alveolar bone defect model

Male SD rats (8-week-old) were purchased from Shanghai Bikai Keyi Biotechnology Co., Ltd. All experimental animal protocols received prior ethical clearance from the Institutional Animal Care and Use Committee of Shanghai Ninth People's Hospital, Shanghai Jiao Tong University School of Medicine (Shanghai, China) (Approval No. SH9H-2024-A99-1). 16 male SD rats were randomly divided into four groups: sham surgery, blank control, SF/PCL, and Cur-Sr/SF/PCL. Alveolar bone voids were generated according to a previously reported method [13]. Briefly, after general anesthesia with Zoletil, the palatal gingiva adjacent to the maxillary first molar was incised using a sterile surgical blade. A mucoperiosteal flap was raised to expose the underlying bone. A standardized alveolar bone lesion, approximately 3 mm (length) × 1.5 mm (width) × 2 mm (depth) was drilled into the palatal side of the maxillary first molar using a low-speed dental drill. The EFM was then placed within the defect site. Subsequently, the mucoperiosteal flap was repositioned and surgical sutures were utilized to close the incision and anchor the membrane. At 8 weeks post-surgery, the animals underwent euthanasia. The maxillae were retrieved and preserved in 4% PFA for later morphological and histological assessment.

The harvested maxillae were scanned using a micro-computed tomography (micro-CT) system (SkyScan1276, Bruker, USA) to evaluate alveolar bone regeneration. The scanning parameters were set at 87 kV and 200 μA, achieving a voxel size of 15 μm. Three-dimensional (3D) and two-dimensional (2D) representations of the alveolar bone were reconstructed and evaluated. The distance between the cementoenamel junction (CEJ) to the alveolar bone crest (ABC) was measured, and the bone volume/tissue volume (BV/TV) ratio within the defect area was calculated. Following micro-CT analysis, the bone specimens were decalcified, embedded, and sectioned for histological processing. Hematoxylin and eosin (H&E) staining, Masson's trichrome staining, as well as immunofluorescence staining for osteopontin (OPN), VEGF, inducible nitric oxide synthase (iNOS) and mannose receptor (CD206) were subsequently performed. All antibodies used are detailed in Table S3.

### 2.17 Statistical analysis

Statistical significance was determined by one-way analysis of variance (ANOVA) with *∗P* < 0.05, *∗∗P* < 0.01, and *∗∗∗P* < 0.001 considered significant (n = 3).

## 3 Results and discussion

### 3.1 Synthesis and characterization of the Cur-Sr NPs

**Fig. 1:**
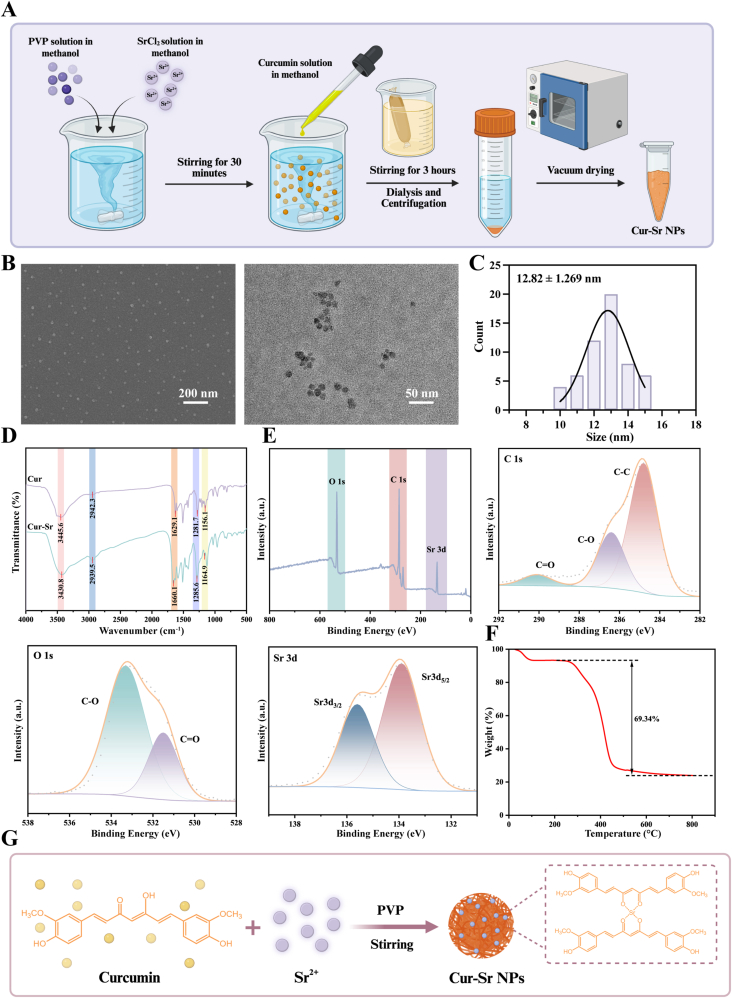
Synthesis and characterization of the Cur-Sr NPs. A) Synthesis procedure of the Cur-Sr NPs. B) SEM and TEM images of the Cur-Sr NPs. C) Diameter distribution of the Cur-Sr NPs. D) FTIR spectra of Cur and the Cur-Sr NPs. E) Full scan XPS survey spectrum and the high-resolution spectra for C 1s, O 1s and Sr 3d of the Cur-Sr NPs. F) TGA of the Cur-Sr NPs. G) Diagram illustrating the mechanism of Cur-Sr NPs synthesis.

We successfully synthesized Cur-Sr NPs based on the chelating ability of curcumin with metal ions. A curcumin-methanol solution was mixed with a strontium salt solution under stirring at room temperature, while PVP was introduced simultaneously. The final product underwent dialysis against deionized water for 48 h to ensure the removal of unbound Sr^2+^, curcumin, and PVP (Fig. 1A).

The physical structure and dimensions of the Cur-Sr NPs were investigated by SEM and TEM. As observed, Cur-Sr NPs possessed a globular shape with a rough surface, having an average diameter measuring approximately 12 nm (Fig. 1B and C). FTIR and XPS were further employed to probe the chemical structure of the Cur-Sr NPs. As shown in Fig. 1D, compared with pure curcumin, the H-O stretching vibration peak of the Cur-Sr NPs between 1150 and 1200 cm^−1^ significantly decreased, suggesting that Sr^2+^ coordinated with the hydroxyl groups in curcumin. The peak observed at 3430 cm^−1^ was assigned to the O-H stretching vibration originating from curcumin's hydroxyl groups. Furthermore, the peak appearing at 1660 cm^−1^ was ascribed to the C=O stretching vibration, as documented previously, validating the presence of PVP within the Cur-Sr NPs [50]. The signal at 1285 cm^−1^ arose from the C-N stretching vibration of PVP, and the signal at 2939 cm^−1^ was assigned to the alkane chain C-H stretching from PVP. The XPS results confirmed the presence of C 1s, O 1s, and Sr 3d signals within the Cur-Sr NPs. The high-resolution C 1s spectrum revealed three individual

components, corresponding to the respective functional groups of C-C, C-O, and C=O. The high-resolution Sr 3d spectrum displayed two peaks at 133.9 eV and 135.7 eV, attributable to Sr 3d_5/2_ and Sr 3d_3/2_, respectively (Fig. 1E). TGA demonstrated that the organic component content in the Cur-Sr NPs reached 69.34% (Fig. 1F). In summary, this study successfully synthesized Cur-Sr NPs by leveraging the ability of curcumin to coordinate with Sr^2+^. The incorporation of PVP served as a stabilizer to control nanoparticle nucleation and growth, yielding monodisperse nanoparticles with excellent colloidal stability. Consequently, Cur-Sr NPs are anticipated to offer superior bioavailability and a controlled release profile over free curcumin. A schematic illustration was provided to elucidate the synthesis mechanism of the Cur-Sr NPs (Fig. 1G).

### 3.2 Synthesis and characterization of the EFMs

**Fig. 2:**
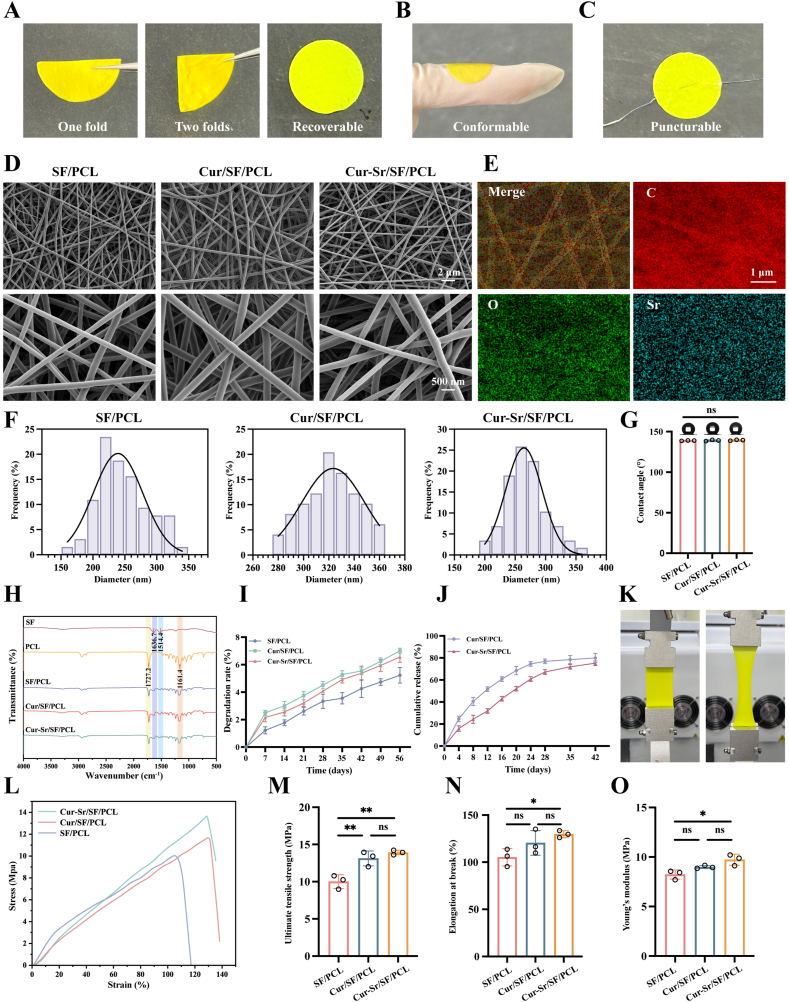
Characterization of the EFMs. A-C) Optical photographs exhibiting the foldability, conformability, and puncturable property of the EFMs. D) SEM images of the EFMs E) Elemental mapping microscopy images of the Cur-Sr/SF/PCL EFM. F) Fiber diameter distribution of the EFMs. G) Water contact angle measurements on the EFMs. H) FTIR spectra of SF, PCL and the EFMs. I) The degradation rates of the EFMs. J) The cumulative release of curcumin from the EFMs. K) Optical photographs of the tensile test of the EFMs. L-O) The stress-strain curve, the ultimate tensile strength, the elongation at break and the Young's modulus of the EFMs. Data are presented as mean ± SD. *∗P* < 0.05, *∗∗P* < 0.01, and *∗∗∗P* < 0.001; ns, not significant.

As shown in Fig. 2A, the Cur-Sr/SF/PCL EFM exhibited excellent foldability, with the capacity to completely regain its initial shape after wetting. When applied to a finger, the membrane demonstrated remarkable conformability, adapting precisely to the contours of the underlying surface (Fig. 2B). Furthermore, during suturing experiments using a suture needle, the membrane allowed easy penetration without exhibiting significant cracking or tearing (Fig. 2C). These tests were designed to simulate critical clinical scenarios encountered in GBR, where membranes are often subjected to folding, cutting, suturing, and must adapt to the irregular morphology of alveolar bone defects. The findings collectively suggested that the Cur-Sr/SF/PCL EFM possessed sufficient flexibility, shape adaptability, suture penetrability, and crack resistance, underscoring its strong potential for clinical applications.

The external architecture of the EFMs, including SF/PCL, Cur/SF/PCL, and Cur-Sr/SF/PCL, was examined using SEM (Fig. 2D, Fig. S1). The results revealed that every fiber was randomly arranged, possessed a consistent diameter and smooth surface, and was devoid of any beaded structures. The overall morphology remained unchanged following the incorporation of the drugs. The average fiber diameter of the various membranes ranged from approximately 250 nm to 350 nm (Fig. 2F). The

introduction of both curcumin and Cur-Sr NPs modestly augmented the fiber diameter. Energy-dispersive X-ray spectroscopy (EDS) mapping confirmed that carbon (C), oxygen (O), and strontium (Sr) elements were evenly spread across the Cur-Sr/SF/PCL EFM, which supported the successful incorporation of Cur-Sr NPs (Fig. 2E).

The results of water contact angle measurements showed that all groups of membranes exhibited hydrophobic characteristics, which can be attributed to the inherent hydrophobicity of PCL (Fig. 2G) [7]. Furthermore, there were no statistically meaningful variations in the water contact angles among different groups. However, incorporation of curcumin and Cur-Sr NPs failed to produce a substantial enhancement of the membranes’ surface wettability. The chemical composition and distinctive functional groups of the EFMs were analyzed via FTIR spectroscopy (Fig. 2H). The characteristic peaks appearing at 1161 cm^−1^ and 1727 cm^−1^ represented the C-O-C and C=O stretching vibrations of PCL, respectively [13,26]. Meanwhile, the peaks at 1636 cm^−1^ and 1514 cm^−1^ were characteristic of the amide I and amide II bands of SF [33]. These findings demonstrated that SF and PCL were successfully co-electrospun. Furthermore, the FTIR spectra of all groups of EFMs exhibited similar surface chemical profiles, implying that the inclusion of curcumin and Cur-Sr NPs did not alter the surface chemistry of the SF/PCL EFM.

We evaluated the biodegradability of the EFMs by measuring their degradation rates. Fig. 2I illustrated that the degradation rate of the membranes did not exceed 4% within 4 weeks and remained within 8% over 8 weeks. Compared to the SF/PCL group, the incorporation of curcumin and Cur-Sr NPs resulted in a faster degradation rate, which could be explained by the increased porosity of the membranes following drug loading. In contrast to the gold-standard Bio-Gide^Ⓡ^ membrane, which lost structural integrity and underwent fragmentation after only 5 days of *in vitro* degradation as previously reported, the Cur-Sr/SF/PCL EFM exhibited significantly enhanced resistance to degradation [17]. Given that the bone defect remodeling phase often persists for one month or longer, the degradation profile of the Cur-Sr/SF/PCL EFM is therefore well-matched with the critical time window required for bone regeneration [51]. The *in vitro* drug release rates of the EFMs were evaluated (Fig. 2J). The cumulative drug release rate for both groups reached approximately 70% after 42 days. Notably, in contrast to the Cur/SF/PCL EFM, the Cur-Sr/SF/PCL EFM exhibited a more sustained, gradual, and prolonged release profile, demonstrating the controlled-release capability of the Cur-Sr NPs.

The ideal GBR membrane should possess sufficient strength, toughness, and ductility to withstand clinical surgical manipulation, endure external pressures arising from oral activities, and accommodate internal stresses generated during tissue regeneration. Therefore, mechanical properties of various EFMs were assessed (Fig. 2K–O). The results revealed that the ultimate tensile strength values for the SF/PCL, Cur/SF/PCL, and Cur-Sr/SF/PCL EFMs were 10.02 ± 0.92 MPa, 13.12 ± 1.00 MPa, and 13.92 ± 0.30 MPa, respectively. The elongation at break values for each group were 105.2 ± 9.27%, 120.4 ± 13.08%, and 129.7 ± 3.67%, while the Young's modulus values were 8.22 ± 0.46 MPa, 8.98 ± 0.15 MPa, and 9.73 ± 0.55 MPa, respectively. In contrast to the Bio-Gide^Ⓡ^ membrane (ultimate tensile strength ≈0.74 MPa; elongation at break ≈29.91 ± 8.58 %) as reported previously, the Cur-Sr/SF/PCL EFM demonstrated significantly enhanced mechanical properties [17]. These robust characteristics are critical for ensuring the membrane's stability during clinical handling and its ability to offer space maintenance for the bone defect area.

### 3.3 Cytocompatibility and barrier function of the EFMs

**Fig. 3:**
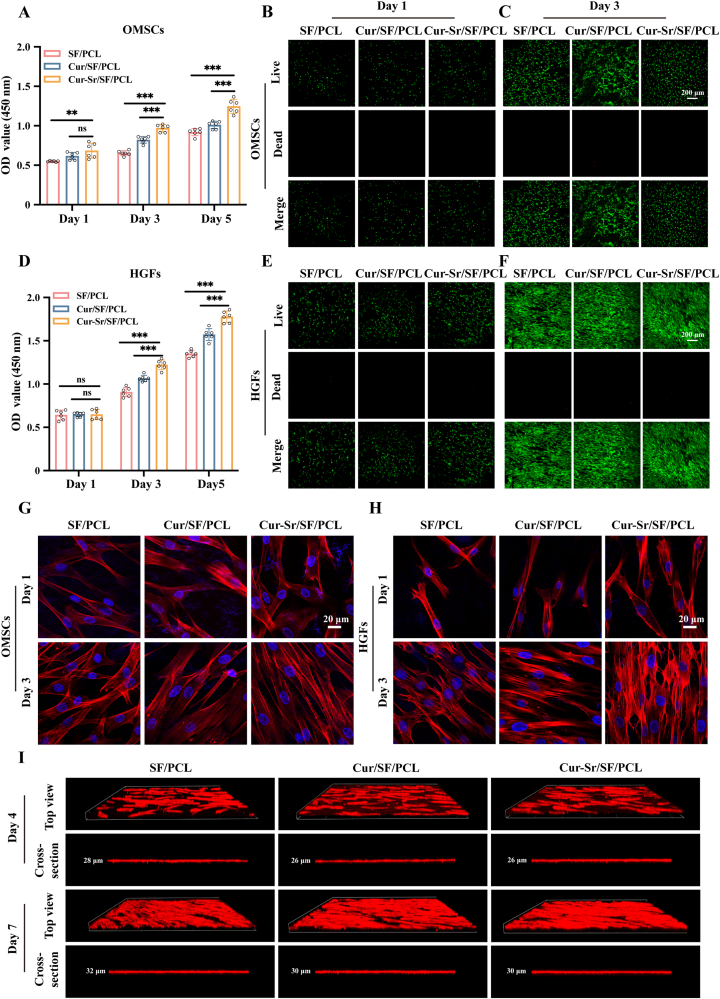
Cytocompatibility and barrier function of the EFMs. A, D) The CCK-8 assay of OMSCs and HGFs maintained on the EFMs at 1, 3 and 5 days post-seeding, respectively. B, C, E, F) Live/dead viability staining of OMSCs and HGFs maintained on the EFMs at 1 and 3 days post-seeding, respectively. G, H) Representative fluorescence micrographs showing OMSCs and HGFs maintained on the EFMs at 1 and 3 days post-seeding, respectively. I) Reconstructed fluorescence micrographs and corresponding cross-sectional views for HGFs maintained on the EFMs at 4 and 7 days post-seeding, respectively. Data are presented as mean ± SD. *∗P* < 0.05, *∗∗P* < 0.01, and *∗∗∗P* < 0.001; ns, not significant.

As has been established, the prerequisite for an ideal GBR membrane is excellent biocompatibility with both soft and hard tissues [10,11]. Consequently, the effect of EFMs incorporating different concentrations of Cur-Sr NPs on cellular propagation was first evaluated. The concentration of Cur-Sr NPs was optimized at 1 mg/mL (Fig. S2). Subsequently, *in vitro* biocompatibility of the SF/PCL, Cur/SF/PCL, and Cur-Sr/SF/PCL EFMs towards OMSCs and HGFs was further assessed. The CCK-8 results demonstrated that at both the 3-day and 5-day time points, the cellular viability of OMSCs and HGFs cultured on the Cur-Sr/SF/PCL EFM was markedly greater than the others, respectively (Fig. 3A and D). This suggested that the Cur-Sr/SF/PCL EFM possessed the strongest capability to promote cell proliferation. OMSCs (Fig. 3B and C) and HGFs (Fig. 3E and F) were subjected to a live/dead viability assay and the findings revealed robust growth for both OMSCs and HGFs on all groups, with a large population of viable cells within the visual field. The scarcity of dead cells indicated that none of the EFMs exhibited cytotoxic effects toward either cell type. Furthermore, nuclei and cytoskeleton staining were conducted after 1 and 3 days of culture (Fig. 3G and H). The results revealed that both OMSCs and HGFs seeded on the EFMs exhibited favorable morphology and uniform distribution, confirming that none of the membranes had a negative impact on the adhesion of either cell type. In conclusion, all three groups of the EFMs exhibited good biocompatibility with OMSCs and HGFs, with the Cur-Sr/SF/PCL EFM distinguished by its optimal performance in promoting cell proliferation, thus satisfying the criteria for an ideal GBR membrane.

Barrier function is an indispensable property of GBR membranes, as it impedes soft tissue from invading the osseous void, thereby creating adequate room for osteoblast proliferation and subsequent bone formation [8]. In this study, CLSM was employed to assess whether HGFs could infiltrate into the membranes (Fig. 3I). The reconstructed fluorescent 3D images and cross-sectional views revealed that although HGFs proliferated rapidly on the EFM surface during 7 days of culturing, the depth of cell distribution showed little change over time. Furthermore, on day 7, the cell distribution depth on the EFMs was approximately 30 μm, which was less than the membrane thickness of 60 μm. These results

indicated that the EFMs possessed a favorable barrier function, capable of resisting soft tissue infiltration in GBR applications.

### 3.4 *In vitro* osteogenic induction capacity of the EFMs

**Fig. 4:**
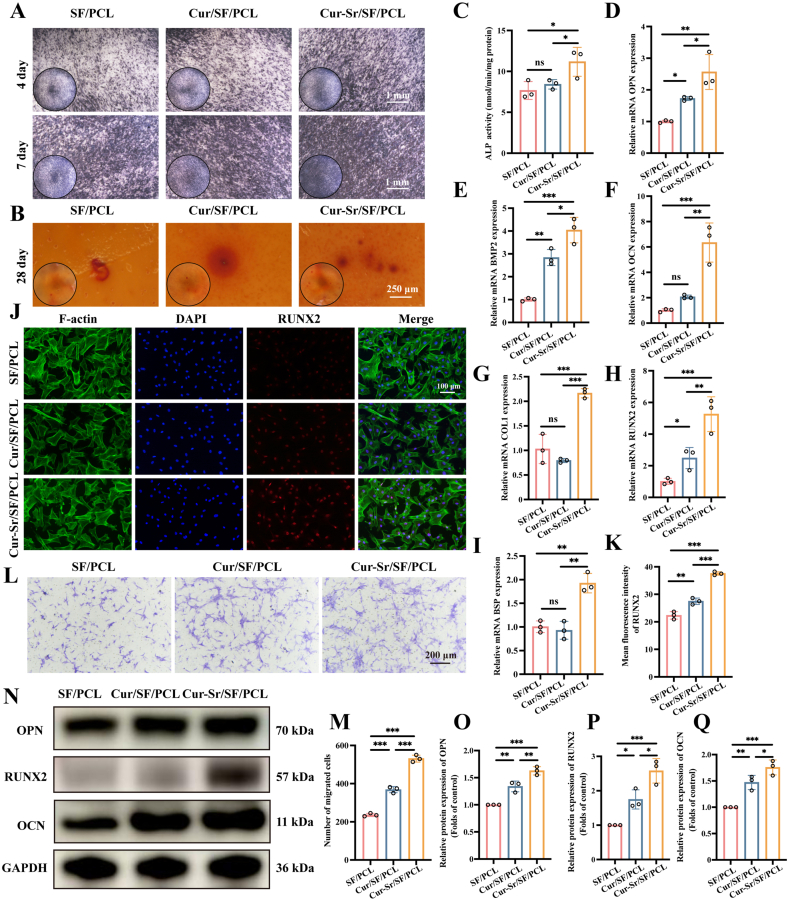
*In vitro* osteogenic induction capacity of the EFMs. A) ALP staining of OMSCs cultured on the EFMs for 4 and 7 days. B) ARS staining of OMSCs cultured on the EFMs for 28 days. C) ALP activity of OMSCs cultured on the EFMs for 7 days. D-I) Relative mRNA expression of osteogenesis-related genes (OPN, BMP2, OCN, COL1, RUNX2, BSP) of OMSCs cultured on the EFMs for 7 days. J, K) The immunofluorescence images and quantitative results of osteogenesis-related protein (RUNX2) of OMSCs cultured on the EFMs for 7 days. L, M) The transwell migration assay and quantitative analysis of OMSCs with the EFMs in the bottom chambers for 24 h. N-Q) Western blot assay and quantitative analysis of the protein expression levels of OPN, RUNX2 and OCN of OMSCs cultured on the EFMs for 14 days. Data are presented as mean ± SD. *∗P* < 0.05, *∗∗P* < 0.01, and *∗∗∗P* < 0.001; ns, not significant.

Experiments were performed to investigate the effect of the EFMs on osteogenic differentiation of OMSCs. OMSCs were co-cultured with the EFMs incorporating varying concentrations of Cur-Sr NPs. ALP staining was conducted to detect ALP level and to screen for the optimal nanoparticle concentration. The results demonstrated that the Cur-Sr NPs (1 mg/mL) group exhibited the most intense positive ALP staining (Fig. S3), confirming that the optimal concentration of Cur-Sr NPs was 1 mg/mL. Subsequently, ALP staining was continued on the SF/PCL, Cur/SF/PCL, and Cur-Sr/SF/PCL EFMs. The results revealed that although the Cur/SF/PCL group showed improved performance over the SF/PCL group, the Cur-Sr/SF/PCL group had a significantly enhanced pro-osteogenic capacity in comparison with the other two groups, and this enhancement was verified by quantitatively analyzing ALP activity at day 7 (Fig. 4A and C). At 28 days post-seeding of OMSCs onto the membranes, we conducted ARS staining to assess the development of mineralized calcium nodules. As shown by the results, while the Cur/SF/PCL group outperformed the SF/PCL control, the Cur-Sr/SF/PCL EFM produced the highest number of mineralized nodules (Fig. 4B).

The expression of osteogenesis-related genes was examined to further validate the pro-osteogenic effect of the EFMs. The genes analyzed included OPN, RUNX2, bone morphogenetic protein 2 (BMP2), osteocalcin (OCN), collagen type I (COL1), and bone sialoprotein (BSP). The results showed that although the Cur/SF/PCL group upregulated the expressions of these genes compared to the SF/PCL group, the Cur-Sr/SF/PCL EFM induced a more substantial upregulation in the expression of these genes in OMSCs, providing further evidence that the inclusion of Cur-Sr NPs stimulated osteogenic differentiation of OMSCs (Fig. 4D–I).

Subsequently, the influence of the EFMs on osteogenic differentiation was examined at the protein level using immunofluorescence staining and Western blot analysis. After culturing OMSCs on the various membranes for 7 days, immunofluorescence staining was performed for RUNX2, a key transcription factor for osteogenic differentiation. The results demonstrated that although the Cur/SF/PCL EFM showed enhanced RUNX2 expression compared to the SF/PCL EFM, the highest expression level of RUNX2 protein was observed in the Cur-Sr/SF/PCL EFM (Fig. 4J and K). Consistent with these findings, Western blot analysis further confirmed the protein expression levels of RUNX2, OPN, and OCN, all of which followed a similar trend, yielding the highest expression in the Cur-Sr/SF/PCL group (Fig. 4N–Q).

The recruitment of OMSCs to the bone defect site has been proved to be an essential phase in bone healing [49,52]. Therefore, we evaluated the recruitment capability of the EFMs for OMSCs using a Transwell migration assay. The results indicated that although the Cur/SF/PCL group exhibited enhanced cell migration compared to the SF/PCL control group, the Cur-Sr/SF/PCL group showed a significantly more robust recruitment effect than the other two groups. These findings suggested that the Cur-Sr NPs could enhance the recruitment of OMSCs (Fig. 4L and M).

In summary, our results primarily demonstrated that the incorporation of curcumin effectively promoted the pro-osteogenic potential of the EFMs, which aligned well with previous findings. For instance, Ghorbaninejad et al. reported that curcumin functions as an epigenetic switch to facilitate the differentiation of bone mesenchymal stem cells, specifically by suppressing EZH2 [53]. Similarly, Wei et al. demonstrated that curcumin drives osteogenic differentiation by modulating the lipid raft/GLUT1

axis to switch on bioenergetic channels [35]. Additionally, studies have shown that curcumin-induced mild endoplasmic reticulum stress enhances osteogenic differentiation by upregulating the expression of ATF6 [54]. Notably, compared to curcumin alone, the Cur-Sr NPs significantly enhances the osteoinductive and recruitment capacity of the SF/PCL EFM, demonstrating superior osteogenic potential. Given that Sr^2+^ has been widely documented to possess potent osteogenic activity [46,47], we conclude that the excellent pro-osteogenic performance of the Cur-Sr/SF/PCL EFM can be ascribed to the controlled release profile of therapeutic agents and the synergistic effect between curcumin and Sr^2+^.

### 3.5 *In vitro* angiogenic function of the EFMs

**Fig. 5:**
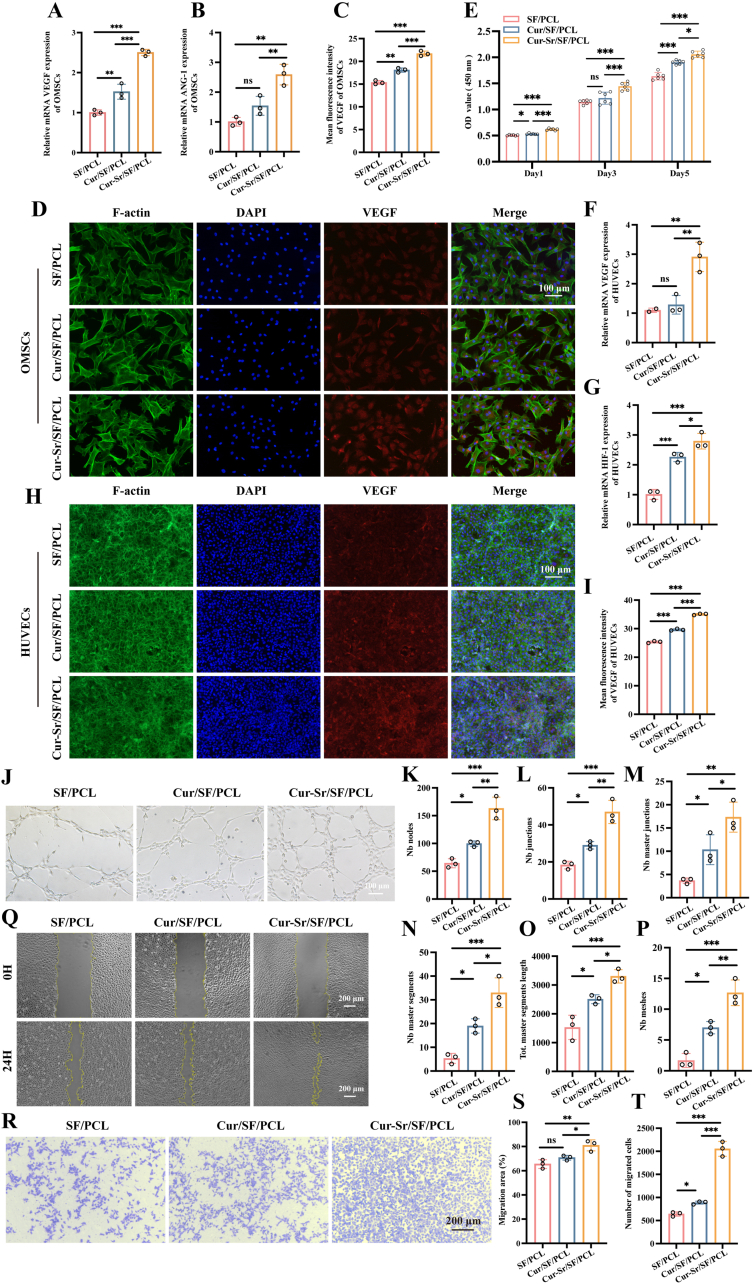
*In vitro* angiogenic function of the EFMs. A, B) Relative mRNA expression of angiogenesis-related genes (VEGF, ANG-1) of OMSCs cultured on the EFMs for 7 days. C, D) The immunofluorescence images and quantitative results of angiogenesis-related protein (VEGF) of OMSCs cultured on the EFMs for 7 days. E) The CCK-8 assay of HUVECs cultured on the EFMs for 1, 3 and 5 days. F, G) Relative mRNA expression of angiogenesis-related genes (VEGF, HIF-1) of HUVECs cultured on the EFMs for 4 days. H, I) The immunofluorescence images and quantitative results of VEGF of HUVECs cultured on the EFMs for 4 days. J-P) The tube formation assay of HUVECs stimulated by the extractions of the EFMs and quantitative results. Q, S) The scratch assay of HUVECs stimulated by the extractions of the EFMs immediately and after 24 h. R, T) The transwell migration assay and quantitative analysis of HUVECs with the EFMs in the bottom chambers for 24 h. Data are presented as mean ± SD. *∗P* < 0.05, *∗∗P* < 0.01, and *∗∗∗P* < 0.001; ns, not significant.

Bone is an extensively vascularized tissue, requiring a robust blood supply to support its repair process. This process is orchestrated by the coupled actions of angiogenesis and osteogenesis. Furthermore, numerous studies have indicated that bone marrow mesenchymal stem cells can secrete various factors to recruit endothelial cells, creating a favorable environment for angiogenesis [27,49]. Therefore, we evaluated the effect of the EFMs on the ability of OMSCs to induce angiogenesis. RT-qPCR results showed that the expression levels of angiogenesis-related genes, including VEGF and angiopoietin-1 (ANG-1), were significantly upregulated in OMSCs co-cultured with the Cur-Sr/SF/PCL EFM (Fig. 5A and B). Immunofluorescence staining for VEGF protein further verified that the Cur-Sr/SF/PCL EFM promoted VEGF protein expression, suggesting its pro-angiogenic function (Fig. 5C and D). According to previous researches, this may be attributed to the synergistic effect of curcumin

and Sr^2+^ in promoting the secretion of pro-angiogenic cytokines by bone marrow mesenchymal stem cells [17,55].

The intrinsic angiogenic capacity of vascular endothelial cells is critical during bone defect repair [49]. Therefore, we first evaluated the biocompatibility of the EFMs with HUVECs via a CCK-8 assay. As shown in Fig. 5E, the Cur-Sr/SF/PCL EFM significantly promoted HUVECs proliferation

compared to the other groups. Using RT-qPCR and immunofluorescence staining, we further detected the expression levels of angiogenesis-associated genes and proteins in HUVECs co-cultured with the Cur-Sr/SF/PCL group. The results revealed marked upregulation of VEGF and hypoxia-inducible factor 1 (HIF-1) genes, along with increased VEGF protein levels in the Cur-Sr/SF/PCL group (Fig. 5F–I). A tube formation assay further confirmed the strong pro-angiogenic capability of the Cur-Sr/SF/PCL EFM, as evidenced by enhanced vascular network formation, including more nodes, junctions, segments, and meshes, as well as greater total master segment length (Fig. 5J–P).

Meanwhile, the recruitment of vascular endothelial cells is vital for enhancing vascularization within the defect area, which in turn facilitates alveolar bone regeneration [27]. Thus, the recruitment of the EFMs towards HUVECs was investigated. Results from the scratch assay showed that the Cur-Sr/SF/PCL group achieved the largest wound closure area for HUVECs over a 24-h period (Fig. 5Q and S). These findings were further corroborated by the Transwell migration assay, which revealed that the Cur-Sr/SF/PCL EFM exhibited the most pronounced pro-migratory activity for HUVECs (Fig. 5R and T).

In summary, the pro-angiogenic capacity of the Cur-Sr/SF/PCL EFM stems from its ability to promote the secretion of pro-angiogenic cytokines by OMSCs and directly enhance the angiogenic potential of HUVECs. This dual mechanism ultimately drives the osteogenic-angiogenic coupling effect, establishing a conducive microenvironment for bone defect repair.

### 3.6 *In vitro* immune reprogramming effect of the EFMs

**Fig. 6:**
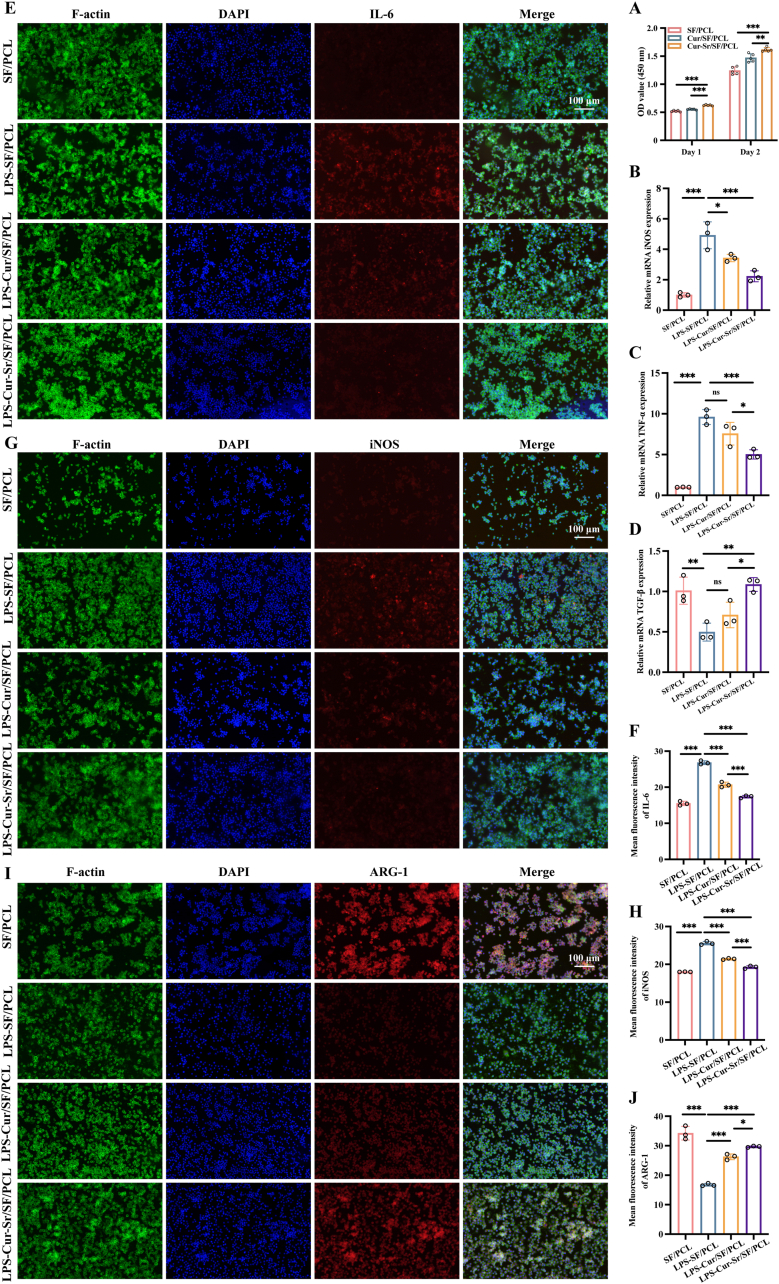
*In vitro* immune reprogramming effect of the EFMs. A) The CCK-8 assay of RAW 264.7 cells cultured on the EFMs for 1 and 2 days. B-D) Relative mRNA expression of M1-related genes (iNOS, TNF-α) and M2-related gene (TGF-β) of RAW 264.7 cells cultured on the EFMs after 24 h of LPS treatment. E-J) The immunofluorescence images and quantitative results of M1-related proteins (iNOS, IL-6) and M2-related protein (ARG-1) of RAW 264.7 cells cultured on the EFMs after 24 h of LPS treatment. Data are presented as mean ± SD. *∗P* < 0.05, *∗∗P* < 0.01, and *∗∗∗P* < 0.001; ns, not significant.

As previously described, following the occurrence of a bone defect, pro-inflammatory M1-type macrophages are rapidly recruited to the defect site and secrete pro-inflammatory cytokines [20]. The persistence of the M1 phenotype without a concomitant enhancement of the M2 phenotype can lead to dysregulation of the immune microenvironment at the bone defect site, ultimately compromising the bone repair outcome. Therefore, an ideal GBR membrane should possess the capability to reprogram macrophages towards the M2 polarization state. To evaluate the immunomodulatory effects of the EFMs, RAW 264.7 cells were co-cultured with the membranes. Results from the CCK-8 assay demonstrated that all membranes exhibited good biocompatibility with RAW 264.7 cells (Fig. 6A). Subsequently, LPS (1 μg/mL) was used to induce M1 polarization in RAW 264.7 cells and the expression levels of genes and proteins associated with polarization were measured in RAW 264.7 cells co-cultured with the EFMs via RT-qPCR and immunofluorescence staining, respectively. As shown in Fig. 6B–D, RT-qPCR results indicated that, compared to the SF/PCL and Cur/SF/PCL groups, the Cur-Sr/SF/PCL EFM significantly suppressed the expression of M1-related genes, including iNOS and tumor necrosis factor-alpha (TNF-α), while promoting the expression of the M2-related gene transforming growth factor-beta (TGF-β). Immunofluorescence staining analysis yielded consistent conclusions, demonstrating that in RAW264.7 cells co-cultured with the Cur-Sr/SF/PCL EFM, the expression of M1-related proteins, iNOS and interleukin-6 (IL-6) was downregulated, while the

expression of the M2-related protein arginase-1 (ARG-1) was upregulated (Fig. 6E–J). These results collectively indicated that the Cur-Sr/SF/PCL EFM possessed the most significant immune reprogramming capacity, proficiently facilitating the shift of macrophages from M1 state towards M2 state. Based on previous literature reports, both curcumin and Sr^2+^ have been confirmed to exhibit immunomodulatory properties [17,49,56]. Consequently, we speculate that the superior immune reprogramming characteristics demonstrated by the Cur-Sr/SF/PCL EFM can be attributed to the synergistic effects stemming from concurrent release of curcumin and Sr^2+^ from the membranes.

### 3.7 The mechanisms of the Cur-Sr/SF/PCL EFM in promoting osteogenesis

**Fig. 7:**
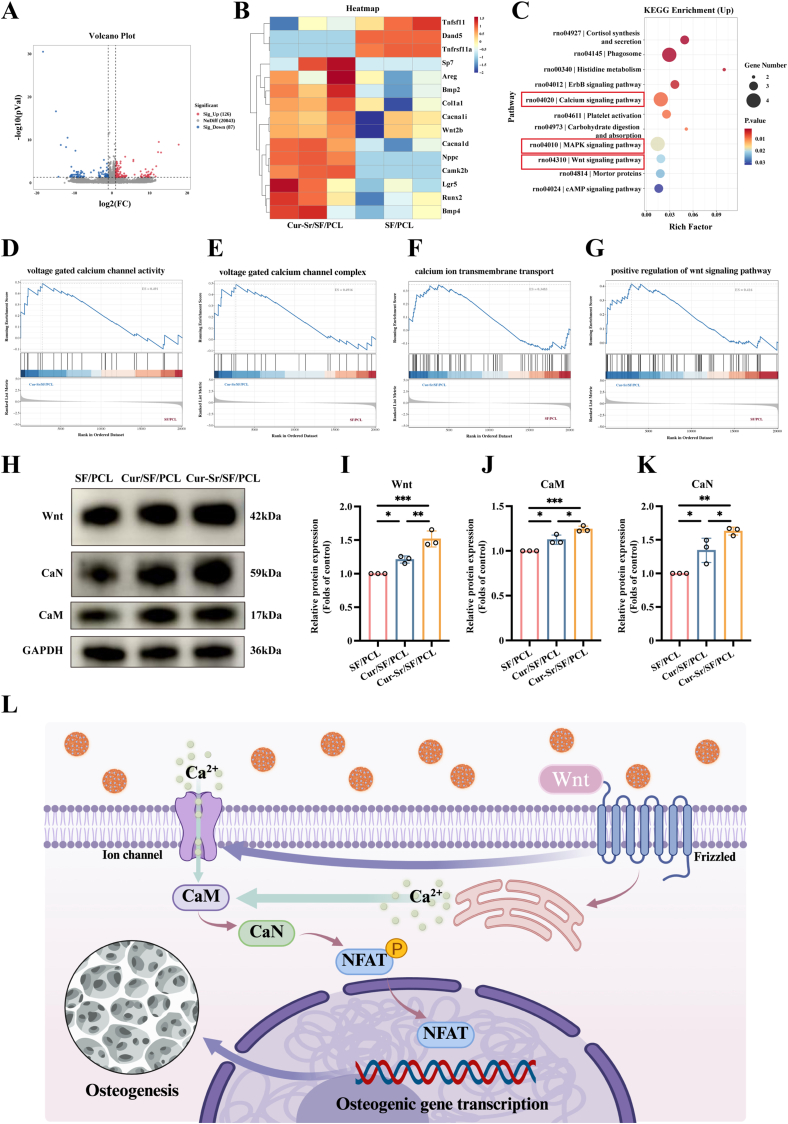
The mechanisms of the Cur-Sr/SF/PCL EFM in promoting osteogenesis based on RNA-seq and analysis. A) The volcano plot of differentially expressed genes between SF/PCL and Cur-Sr/SF/PCL groups. B) The heatmap of differentially expressed genes between SF/PCL and Cur-Sr/SF/PCL groups. C) KEGG enrichment analysis of upregulated differentially expressed genes between SF/PCL and Cur-Sr/SF/PCL groups. D-G) GSEA plots of voltage-gated calcium channel activity, voltage-gated calcium channel complex, calcium ion transmembrane transport and the positive regulation of Wnt signaling pathway between SF/PCL and Cur-Sr/SF/PCL groups. H-K) Western blot assay and quantitative analysis of the protein expression levels of Wnt, CaM and CaN of OMSCs cultured on the EFMs for 24 h. L) Schematic illustration of the Wnt/Ca^2+^/CaN signaling pathway of OMSCs cultured on the Cur-Sr/SF/PCL EFM. Data are presented as mean ± SD. *∗P* < 0.05, *∗∗P* < 0.01, and *∗∗∗P* < 0.001; ns, not significant.

To further investigate the underlying mechanism by which the Cur-Sr/SF/PCL EFM promotes bone formation, RNA-seq analysis was performed. Given that OMSCs serve as the central executioners of bone regeneration, we prioritized OMSCs for this transcriptomic investigation. Principal component analysis (PCA) showed low intra-group variance and clear inter-group separation, indicating distinct transcriptomic profiles (Fig. S4). The volcano plot identified 126 significantly upregulated genes and 87 downregulated genes in the Cur-Sr/SF/PCL group compared to control groups, suggesting a substantial impact on the global gene expression profile (Fig. 7A). The heatmap analysis revealed that, compared to the SF/PCL control, the Cur-Sr/SF/PCL group exhibited an upregulation of classic osteogenic genes, including Sp7, Runx2, Col1a1, and Bmp2/4. Furthermore, we observed a concurrent upregulation of genes associated with the Calcium signaling pathway (Cacna1d, Cacna1i) and the Wnt signaling pathway (Wnt2b, Lgr5) (Fig. 7B). Kyoto Encyclopedia of Genes and Genomes (KEGG)

pathway enrichment analysis revealed that the Cur-Sr/SF/PCL EFM significantly activated osteogenesis-related signaling pathways, including the Calcium signaling pathway, MAPK signaling pathway, and Wnt signaling pathway (Fig. 7C). Subsequent Gene Set Enrichment Analysis (GSEA) demonstrated upregulation of gene sets related to voltage-gated calcium channel activity, voltage-gated calcium channel complex, and calcium ion transmembrane transport, collectively indicating enhanced calcium ion influx (Fig. 7D–F). Furthermore, the upregulation of the Wnt signaling pathway corroborated the findings from previous analysis (Fig. 7G).

The non-canonical Wnt signaling pathway plays a critical role in the recruitment, maintenance, and osteogenic differentiation of bone marrow mesenchymal stem cells [57]. It has been demonstrated to promote osteogenesis via the Wnt/Ca^2+^/calcineurin (CaN) signaling pathway. Evidence suggests that binding of Wnt proteins to Frizzled (Fz) receptors on the cell membrane leads to phospholipase Cγ (PLCγ) activation. Activated PLCγ increases inositol 1,4,5-trisphosphate (IP_3_) levels, which stimulates calcium release from the endoplasmic reticulum into the cytoplasm via IP_3_ receptors [58]. Alternatively, Wnt-receptor interaction can mediate the opening of calcium channels, promoting calcium influx [59]. The elevated cytoplasmic Ca^2+^ concentration enhances calmodulin (CaM) levels, which subsequently upregulates CaN protein expression. Activated CaN dephosphorylates nuclear factor of activated T-cells (NFAT), facilitating its nuclear translocation and initiating the transcription of osteogenic genes [25,26,60]. Additionally, curcumin has been shown to activate the Wnt/Ca^2+^/CaN pathway [59]. Therefore, integrating our RNA-seq results, we hypothesized that the Cur-Sr/SF/PCL EFM promoted osteogenesis by activating the Wnt/Ca^2+^/CaN signaling pathway. To validate this hypothesis, Western blot analysis was conducted on OMSCs co-cultured with the different EFMs. The findings revealed a significant increase in the relative protein expression of Wnt, CaM, and CaN within the Cur-Sr/SF/PCL group, confirming the activation of the Wnt/Ca^2+^/CaN pathway (Fig. 7H–K). Moreover, Sr^2+^ has been proven to promote osteogenesis through the activation of the calcium-sensing receptor (CaSR), thereby exerting a synergistic effect with curcumin [49]. A schematic diagram summarizing the proposed mechanism by which Cur-Sr NPs promote osteogenic differentiation via the Wnt/Ca^2+^/CaN signaling pathway is presented in Fig. 7L.

### 3.8 *In vivo* therapeutic effect of the EFMs in a rat alveolar bone defect model

**Fig. 8:**
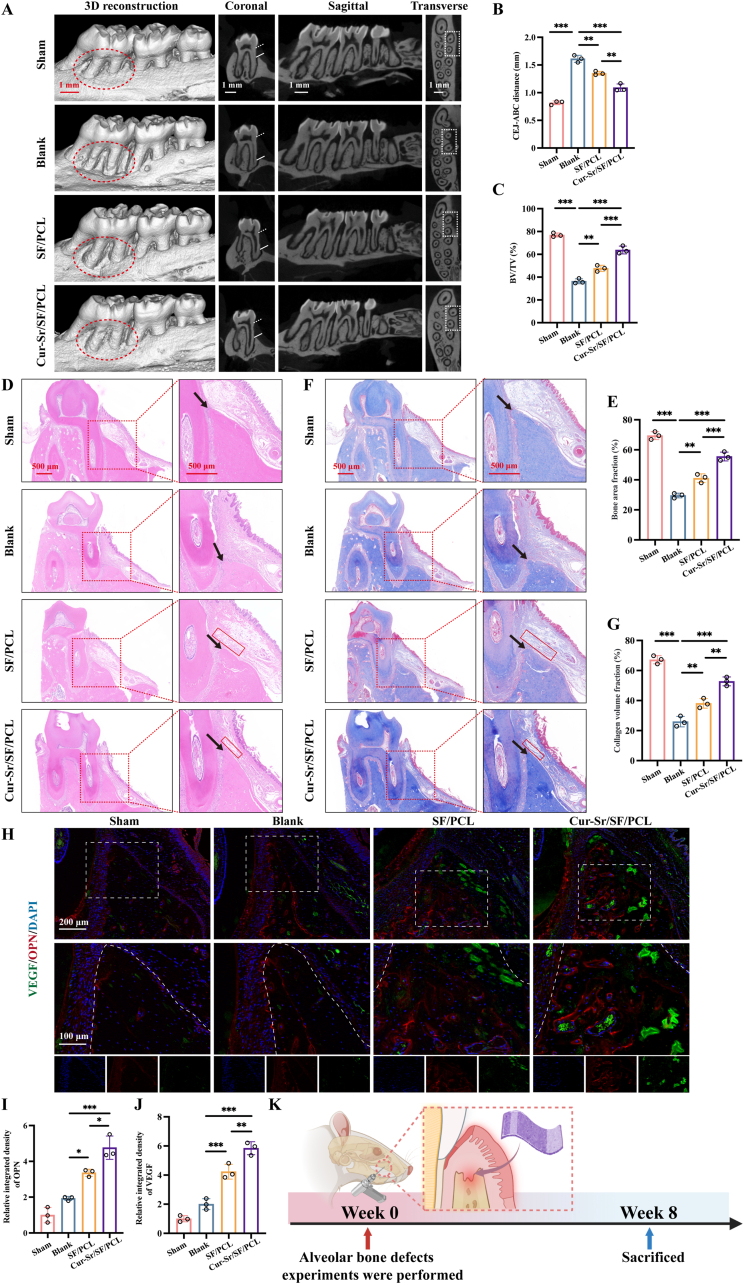
*In vivo* therapeutic effect of the EFMs in a rat alveolar bone defect model. A) 3D reconstruction and sectioned views of the rat maxillary alveolar bones. B, C) Quantitative results calculated from micro-CT data: CEJ-ABC distance, BV/TV. D, E) H&E staining and quantitative analysis. F, G) Masson's trichrome staining and quantitative analysis. H-J) The immunofluorescence images and quantitative results of OPN and VEGF. K) Schematic illustration depicting the animal model establishment and the experimental process. Data are presented as mean ± SD. *∗P* < 0.05, *∗∗P* < 0.01, and *∗∗∗P* < 0.001; ns, not significant.

Prompted by the excellent biological activities of the Cur-Sr/SF/PCL EFM observed in previous *in vitro* experiments, we further evaluated its *in viv*o osteogenic efficacy. The EFMs were surgically placed in the alveolar bone voids of rats, and maxillary samples were collected 8 weeks after the procedure (Fig. 8K). Micro-CT 3D reconstruction results revealed significant bone loss at the palatal aspect of the maxillary first molar in Blank group (Fig. 8A, red circle), confirming the successful creation of the model. Conversely, material implantation groups, particularly the Cur-Sr/SF/PCL EFM, demonstrated a substantial enhancement of bone volume within the defect area. Further observation and quantitative analysis were performed on 2D sections. Coronary images showed that the distance between the CEJ (Fig. 8A, white dashed line) and the ABC (Fig. 8A, white solid line) was markedly increased in Blank group relative to Sham group, while material implantation groups (SF/PCL and Cur-Sr/SF/PCL) showed notable restoration of alveolar ridge height. The Cur-Sr/SF/PCL group demonstrated the most pronounced reduction in the CEJ-ABC distance, indicating its superior capability for repairing alveolar bone defects (Fig. 8B). Consistent results were observed in the transverse sections (white rectangle). Bone morphometric analysis showed that the bone volume fraction (BV/TV) was notably reduced in Blank group relative to Sham group, whereas the value was substantially increased in the Cur-Sr/SF/PCL group, confirming that the Cur-Sr/SF/PCL EFM effectively stimulated osteogenesis (Fig. 8C).

To further evaluate new bone formation within the defect areas, decalcified samples were analyzed using H&E staining and Masson's trichrome staining (Fig. 8D–G). Histological analysis indicated a marked decrease in alveolar ridge height in Blank group in comparison to Sham group, whereas groups implanted with the materials (SF/PCL and Cur-Sr/SF/PCL) demonstrated varying degrees of height recovery (Fig. 8D and F, black arrow). Notably, the Cur-Sr/SF/PCL group exhibited the most robust bone defect repair, presenting the greatest new bone volume and the most mature collagenous matrix. The quantitative results revealed that the Cur-Sr/SF/PCL group achieved the highest new bone area fraction and collagen volume fraction, which is consistent with the Micro-CT data (Fig. 8E and G). Furthermore, minimal residual electrospun fibers were observed within the defect area (Fig. 8D and F, red rectangle), indicating that the degradation rate of the EFMs was synchronized with the bone repair process, thus providing sustained physical support, effective barrier function, and desired biological outcomes throughout the repair period. *In vivo* osteogenic and angiogenic capacities were further investigated by immunofluorescence staining for OPN and VEGF. The results revealed that the most intense fluorescence signals for both OPN and VEGF were detected in the region of new bone in Cur-Sr/SF/PCL group (Fig. 8H–J). These findings have suggested that the Cur-Sr/SF/PCL EFM potently enhances bone formation and vascularization synergistically, thereby corroborating the findings from previous *in vitro* experiments. Furthermore, we extended our investigation to evaluate the *in vivo* immunomodulatory effects of the EFMs (Fig. S5). Immunofluorescence staining results revealed that, compared to the Blank and SF/PCL groups, the Cur-Sr/SF/PCL group exhibited the lowest fluorescence intensity for iNOS and the highest intensity for CD206 within the bone defect regions. These findings confirm that the Cur-Sr/SF/PCL EFM effectively regulates the local immune

microenvironment by suppressing M1 polarization and promoting M2 polarization, which ultimately supports the synergistic bone formation and vascularization observed above.

## 4 Conclusion

In this study, a multifunctional bioactive EFM loaded with Cur-Sr NPs (Cur-Sr/SF/PCL) was successfully developed for GBR. The Cur-Sr/SF/PCL EFM exhibited favorable mechanical properties, an appropriate degradation rate, and effective soft tissue barrier function. Through the synergistic release of curcumin and Sr^2+^, it exerted multiple biological regulatory effects. *In vitro* experiments confirmed that the material significantly promoted osteogenic differentiation, enhanced angiogenic capacity, and effectively modulated the immune microenvironment, thus accelerating alveolar bone healing. In a rat alveolar bone defect model, the Cur-Sr/SF/PCL EFM demonstrated remarkable bone regeneration outcomes. Mechanistic investigation revealed that its pro-osteogenic potential was likely mediated by the activation of the Wnt/Ca^2+^/CaN signaling pathway.

Notably, compared with existing clinical technologies, such as the gold-standard Bio-Gide® collagen membrane, the Cur-Sr/SF/PCL EFM offers distinct advantages. While Bio-Gide® is highly biocompatible, its rapid degradation and poor mechanical stability in moist environments often lead to the collapse of the membrane into the defect, compromising the space-maintaining capacity required for optimal bone regeneration [17]. In contrast, the Cur-Sr/SF/PCL EFM provides superior mechanical strength and a synchronized degradation rate. More importantly, unlike conventional membranes that primarily serve as inert physical barriers with limited biological activity, the Cur-Sr/SF/PCL EFM designed in this study leverages the synergistic release of bioactive components to effectively promote osteogenesis and angiogenesis while inducing immune reprogramming.

Although the outcomes are promising, several limitations of the present study should be acknowledged. While the rat alveolar bone defect model provided essential preliminary evidence, it cannot fully simulate the complex physiological and mechanical environment of the human oral cavity. Therefore, future investigations using large animal models remain necessary for further clinical validation. Furthermore, the structural design of the EFM could be further optimized. Future efforts may focus on developing Janus membranes with asymmetric structures and distinct functional layers.

In summary, this multifunctional approach represents a significant advancement over traditional GBR membranes. The Cur-Sr/SF/PCL EFM meets the key requirements of an ideal GBR membrane and shows promising application potential in clinical applications for alveolar bone regeneration.

## CRediT authorship contribution statement

**Jia Zhou:** Formal analysis, Investigation, Writing – original draft. **Yue Hu:** Methodology, Project administration, Resources. **Jiali Bao:** Data curation, Software, Validation. **Shiyuan Yang:** Methodology, Software. **Yan Zhu:** Validation. **Zixiao Zhang:** Validation. **Minxi Chen:** Validation. **Yuning Zhou:** Methodology. **Kaili Lin:** Conceptualization, Methodology, Project administration, Writing – review & editing. **Yuanjin Xu:** Conceptualization, Project administration, Supervision, Writing – review & editing.

## Declaration of competing interest

The authors declare that they have no known competing financial interests or personal relationships that could have appeared to influence the work reported in this paper.

## Data Availability

Data will be made available on request.
