# Supplementary material for: Synergistic osteogenesis, angiogenesis, and immune reprogramming by a metal-phenolic functionalized electrospun fibrous membrane for alveolar bone regeneration
Source: Mater Today Bio. 2026 Mar 18;38:103045. doi: 10.1016/j.mtbio.2026.103045 (PMC13049611; doi:10.1016/j.mtbio.2026.103045)
Supplement: Multimedia component 1 [file mmc1.docx]

**Supplementary Materials**

**Synergistic osteogenesis, angiogenesis, and immune reprogramming by a metal-phenolic functionalized electrospun fibrous membrane for alveolar bone regeneration**

Jia Zhou ^a, b, 1^, Yue Hu ^a, b, 1^, Jiali Bao ^a, b, 1^, Shiyuan Yang ^a, b^, Yan Zhu ^a, b^, Zixiao Zhang ^a, b^, Minxi Chen ^a, b^, Yuning Zhou ^a, b^, Kaili Lin ^b, c, **^, Yuanjin Xu ^a, b, *^

*^a^* *Department of Oral Surgery, Shanghai Ninth People’s Hospital, Shanghai Jiao Tong University School of Medicine, Huangpu District, Shanghai, China*

*^b^ College of Stomatology, Shanghai Jiao Tong University, National Center for Stomatology, National Clinical Research Center for Oral Diseases, Shanghai Key Laboratory of Stomatology, Shanghai Research Institute of Stomatology, Huangpu District, Shanghai, China*

*^c^ Department of Oral and Cranio-maxillofacial Surgery, Shanghai Ninth People’s Hospital, Shanghai Jiao Tong University School of Medicine, Huangpu District, Shanghai, China*

^1^ These authors contributed equally to this work.

* Corresponding authors: drxuyuanjin@126.com (Y. Xu), lklecnu@aliyun.com (K. Lin).

**S1-Materials and methods**

**Histological and Immunofluorescence staining**

For histological evaluation, tissue sections were deparaffinized in xylene and rehydrated through a graded ethanol series. Hematoxylin and Eosin (H&E) staining (Absin, abs9217, China) and Masson’s trichrome (Solarbio, G1340, China) staining were then performed strictly according to the manufacturer’s instructions using commercial kits. For immunofluorescence analysis, after deparaffinization, rehydration, heat-induced antigen retrieval, permeabilization, and blocking with BSA, the sections were incubated with specific primary antibodies overnight at 4 °C. The following day, the sections were washed and incubated with corresponding fluorescent secondary antibodies for 1 hour at room temperature in the dark. Finally, the sections were mounted and nuclei were counterstained using an antifade mounting medium containing DAPI. Detailed information regarding all primary and secondary antibodies used in this study is listed in Table S3.

**S2-Supplementary tables and figures**

**Table S1. The detailed electrospinning parameters.**

| **Sample group** | **Solution concentration** | **Drug concentration** | **Solution feed rate** | **Applied voltage** | **Collector rotation speed/Collection distance** |
| --- | --- | --- | --- | --- | --- |
| SF/PCL | 10%, w/v  (SF: PCL=2:8) | / | 0.6 ml/h | ±10 kV | 10rpm/15 cm |
| Cur/SF/PCL | 10%, w/v  (SF: PCL=2:8) | Curcumin  (1 mg/ml) | 0.6 ml/h | ±10 kV | 10rpm/15 cm |
| Cur-Sr/SF/PCL-0.5 | 10%, w/v  (SF: PCL=2:8) | Cur-Sr NPs  (0.5 mg/ml) | 0.6 ml/h | ±10 kV | 10rpm/15 cm |
| Cur-Sr/SF/PCL-1 | 10%, w/v  (SF: PCL=2:8) | Cur-Sr NPs  (1 mg/ml) | 0.6 ml/h | ±10 kV | 10rpm/15 cm |
| Cur-Sr/SF/PCL-2 | 10%, w/v  (SF: PCL=2:8) | Cur-Sr NPs  (2 mg/ml) | 0.6 ml/h | ±10 kV | 10rpm/15 cm |
| Cur-Sr/SF/PCL-4 | 10%, w/v  (SF: PCL=2:8) | Cur-Sr NPs  (4 mg/ml) | 0.6 ml/h | ±10 kV | 10rpm/15 cm |

**Table S2. RT-qPCR primer sequences used in this study.**

| **Gene** | **Species** | **Primer** | **Sequences (5’-3’)** |
| --- | --- | --- | --- |
| OPN | Rat | Forward | CCAAGCGTGGAAACACACAGCC |
|  |  | Reverse | GGCTTTGGAACTCGCCTGACTG |
| BMP2 | Rat | Forward | TGCTTCTTAGACGGACTGCG |
|  |  | Reverse | GGGGAAGCAGCAACACTAGA |
| OCN | Rat | Forward | GCCCTGACTGCATTCTGCCTCT |
|  |  | Reverse | TCACCACCTTACTGCCCTCCTG |
| COL1 | Rat | Forward | CAGGCTGGTGTGATGGGATT |
|  |  | Reverse | CCAAGGTCTCCAGGAACACC |
| RUNX2 | Rat | Forward | ACAACCACAGAACCACAAG |
|  |  | Reverse | TCTCGGTGGCTGGTAGTGA |
| BSP | Rat | Forward | AGAAAGAGCAGCACGGTTGAGT |
|  |  | Reverse | GACCCTCGTAGCCTTCATAGCC |
| ANG-1 | Rat | Forward | AAAGGTCAGAAGAGAGGAGCAAG |
|  |  | Reverse | AAGGAAAACTGTCATTGTACTGCC |
| VEGF | Rat | Forward | CAATGATGAAGCCCTGGAGTG |
|  |  | Reverse | AGGTTTGATCCGCATGATCTG |
| GAPDH | Rat | Forward | CCGCATCTTCTTGTGCAGTG |
|  |  | Reverse | GGTAACCAGGCGTCCGATAC |
| HIF-1 | Human | Forward | GAACGTCGAAAAGAAAAGTCTCG |
|  |  | Reverse | CCTTATCAAGATGCGAACTCACA |
| VEGF | Human | Forward | GGCTCTGAAACCATGAACTTTCT |
|  |  | Reverse | GCAATAGCTGCGCTGGTAGAC |
| GAPDH | Human | Forward | GGCTGTTGTCATACTTCTCATGG |
|  |  | Reverse | GGAGCGAGATCCCTCCAAAAT |
| iNOS | Mouse | Forward | CTGCTGGTGGTGACAAGCACATTT |
|  |  | Reverse | ATGTCATGAGCAAAGGCGCAGAAC |
| TNF-α | Mouse | Forward | CAGGCGGTGCCTATGTCTC |
|  |  | Reverse | CGATCACCCCGAAGTTCAGTAG |
| TGF-β | Mouse | Forward | CTCCCGTGGCTTCTAGTGC |
|  |  | Reverse | GCCTTAGTTTGGACAGGATCTG |
| β-actin | Mouse | Forward | CAGCCTTCCTTCTTGGGTATG |
|  |  | Reverse | GGCATAGAGGTCTTTACGGATG |

**Table S3. Antibodies used in this study.**

| **Antibody and Host** | **Antibody Type** | **Dilution** | **Cat. No. and Source** |
| --- | --- | --- | --- |
| RUNX2 Rabbit mAb | Primary antibody | 1:1000 | ET1612-47, Huabio, China |
| RUNX2 Rabbit mAb | Primary antibody | 1:1000 | 12556, Cell Signaling Technology, USA |
| OCN Rabbit pAb | Primary antibody | 1:1000 | A6205, Abclonal, China |
| VEGF Mouse mAb | Primary antibody | 1:100 | MA5-13182, ThermoFisher, USA |
| OPN Rabbit pAb | Primary antibody | 1:200 | 22952-1-AP, Proteintech, China |
| IL-6 Rabbit mAb | Primary antibody | 1:200 | 12912, Cell Signaling Technology, USA |
| iNOS Rabbit mAb | Primary antibody | 1:600 | 13120, Cell Signaling Technology, USA |
| ARG-1 Rabbit pAb | Primary antibody | 1:200 | 16001-1-AP, Proteintech, China |
| CD206 Rabbit mAb | Primary antibody | 1:500 | 24595, Cell Signaling Technology, USA |
| GAPDH Rabbit pAb | Primary antibody | 1:20000 | 10941-1-AP, Proteintech, China |
| Wnt Rabbit mAb | Primary antibody | 1:2000 | A23997, Abclonal, China |
| Calmodulin Rabbit mAb | Primary antibody | 1:1000 | A4885, Abclonal, China |
| Calcineurin Rabbit mAb | Primary antibody | 1:1000 | A4346, Abclonal, China |
| Goat Anti-Rabbit IgG H&L (Alexa Fluor^®^ 594) | Secondary antibody | 1:800 | ab150080, Abcam, the UK |
| Goat Anti-Rabbit IgG H&L (Alexa Fluor^®^ 488) | Secondary antibody | 1:800 | ab150077, Abcam, the UK |
| Donkey Anti-Mouse IgG H&L (Alexa Fluor^®^ 594) | Secondary antibody | 1:800 | ab150108, Abcam, the UK |
| Goat Anti-Mouse IgG H&L (Alexa Fluor^®^ 488) | Secondary antibody | 1:800 | ab150113, Abcam, the UK |
| HRP-conjugated Goat Anti-Rabbit IgG(H+L) | Secondary antibody | 1:5000 | SA00001-2, Proteintech, China |

**Figure S1. SEM images of the EFMs.**


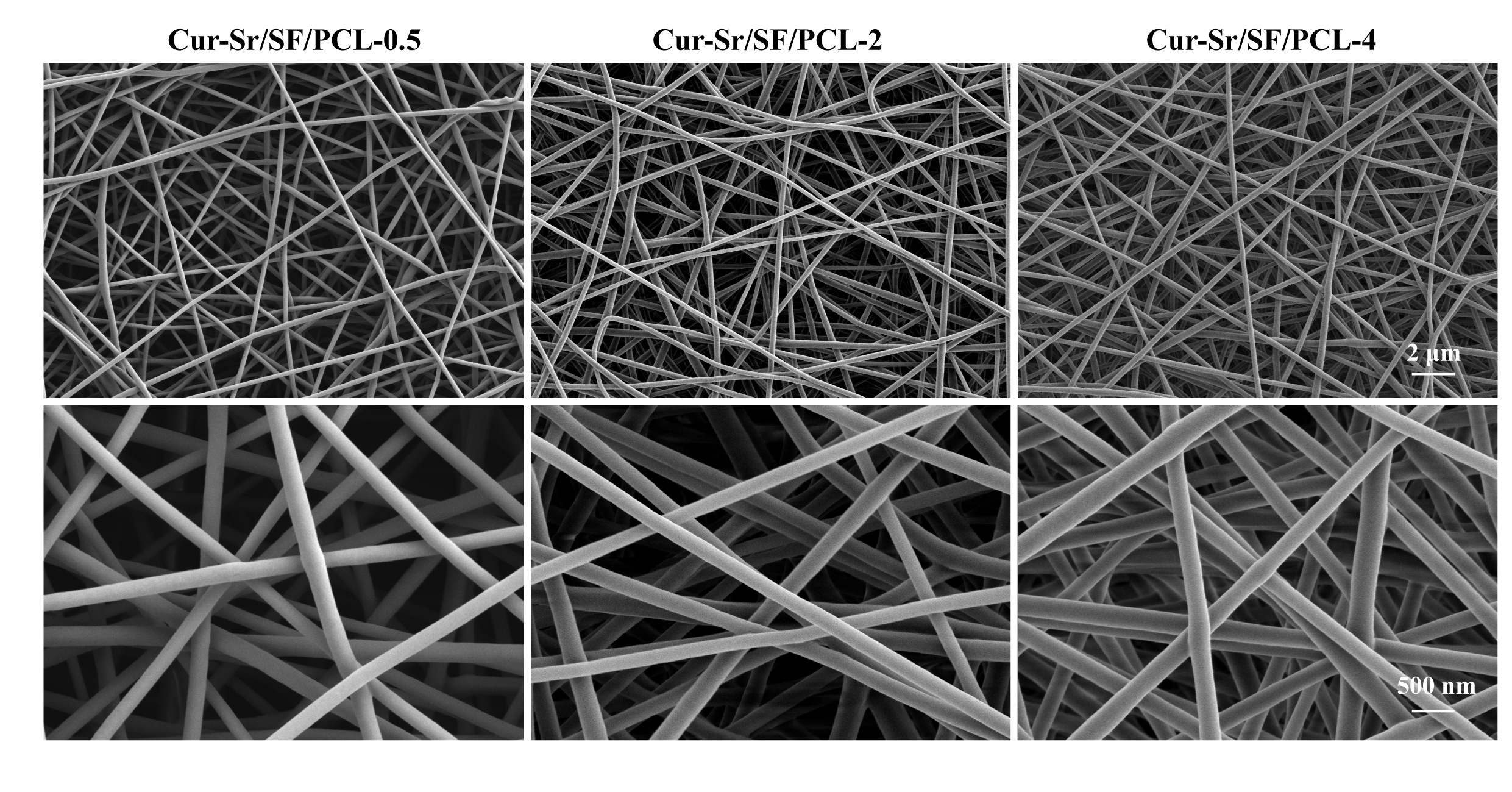


**
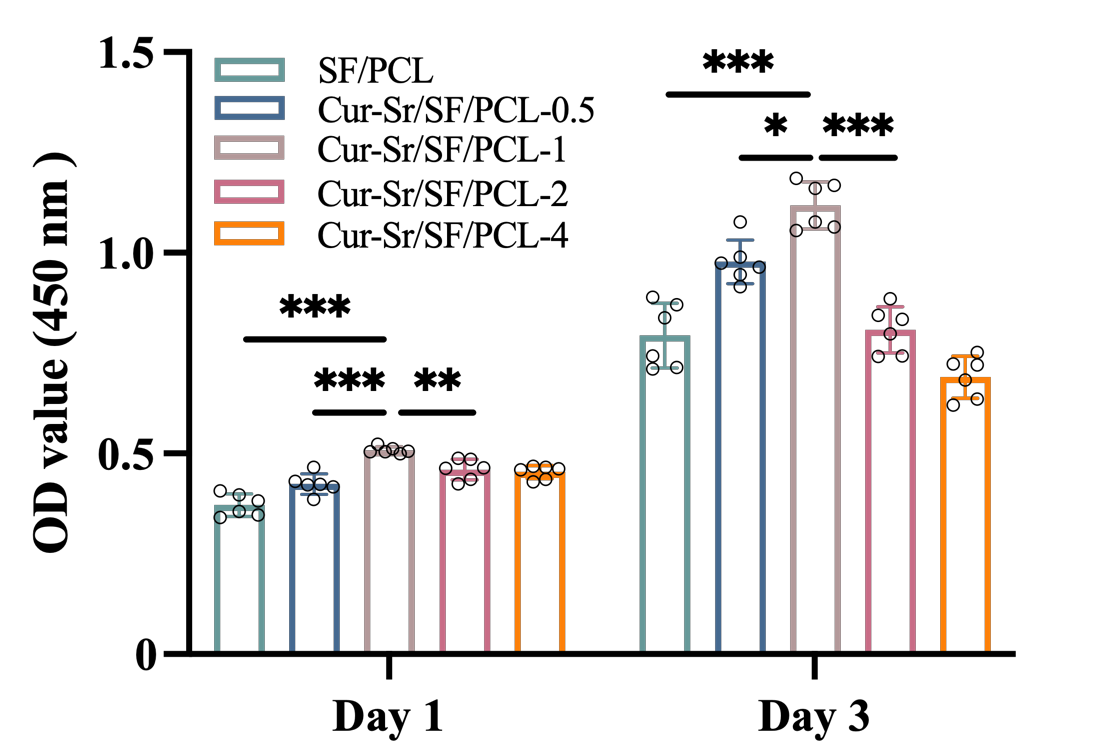
Figure S2. Cytocompatibility of the EFMs containing different concentrations of Cur-Sr NPs.**

**Figure S3. *In vitro* osteogenic induction capacity of the EFMs containing different concentrations of Cur-Sr NPs.**

**
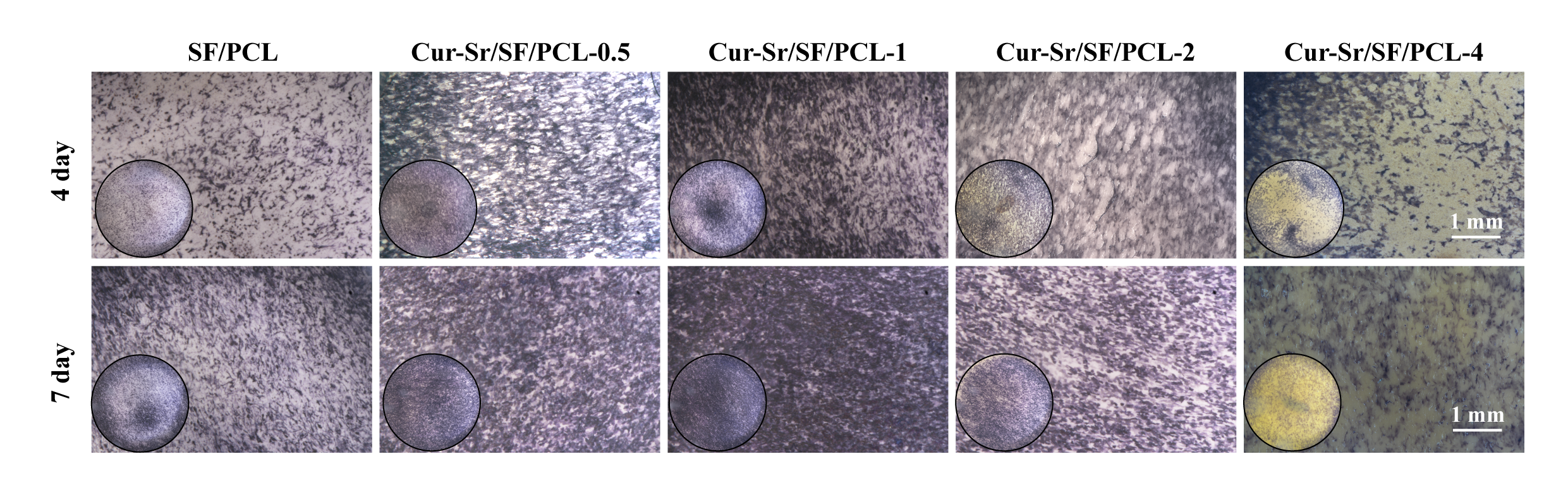
**

**Figure S4. PCA showed PC1 and PC2 for all RNA-seq data of SF/PCL and Cur-Sr/SF/PCL groups.**

**
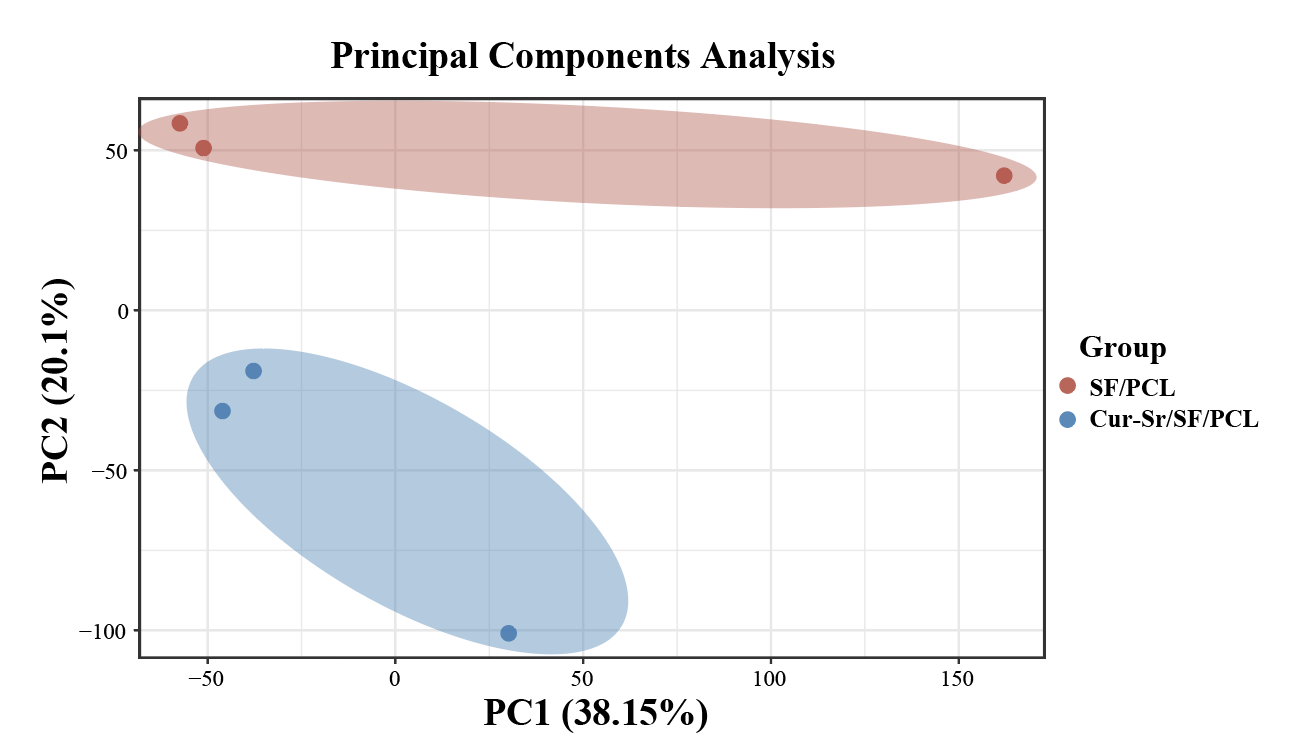
**

**Figure S5. *In vivo* immunomodulatory effects of the EFMs.**


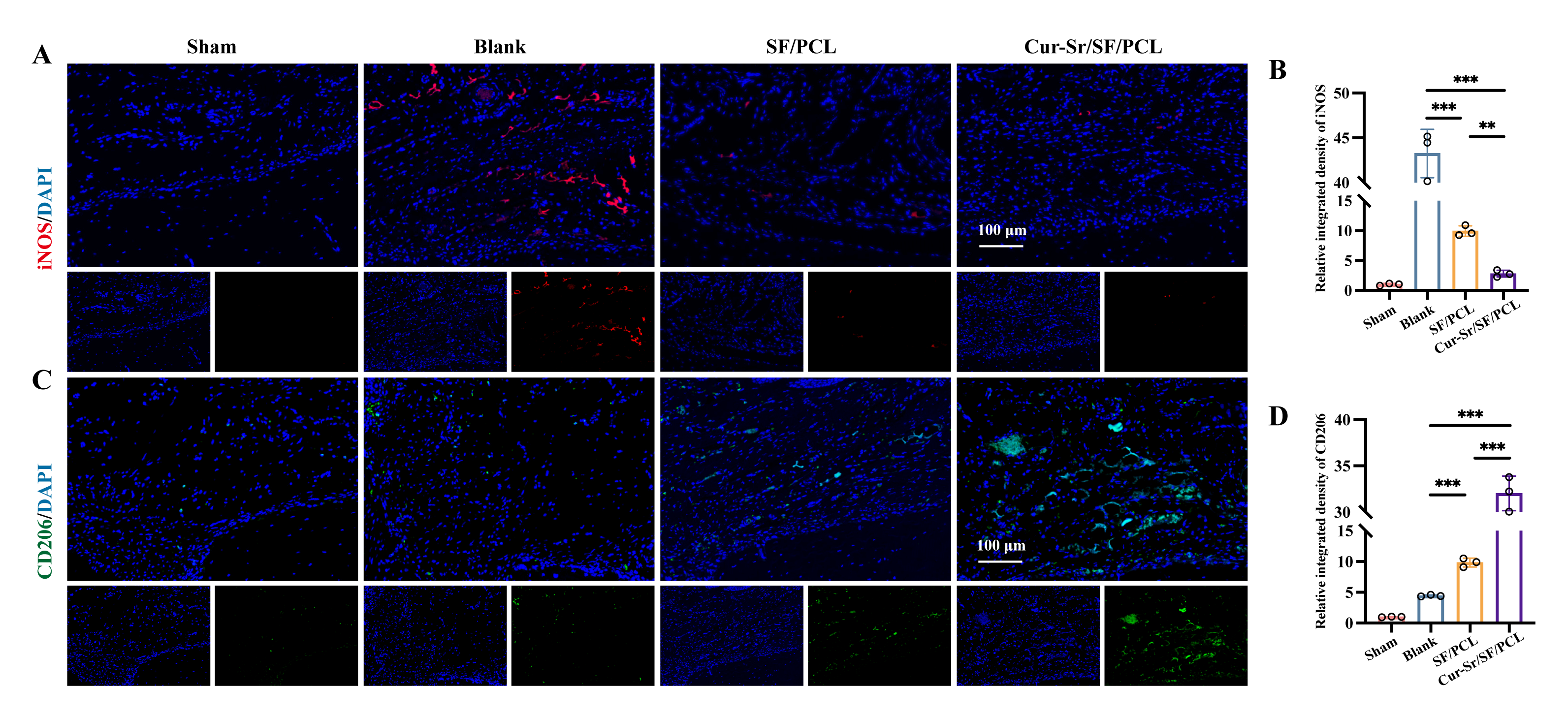


**A, B) The immunofluorescence images and quantitative results of iNOS. C, D) The immunofluorescence images and quantitative results of CD206**
